# Supplementary figures and images for: Genome-wide association studies of seven agronomic traits under two sowing conditions in bread wheat
Source: BMC Plant Biol. 2019 Apr 17;19:149. doi: 10.1186/s12870-019-1754-6 (PMC6475106; doi:10.1186/s12870-019-1754-6)

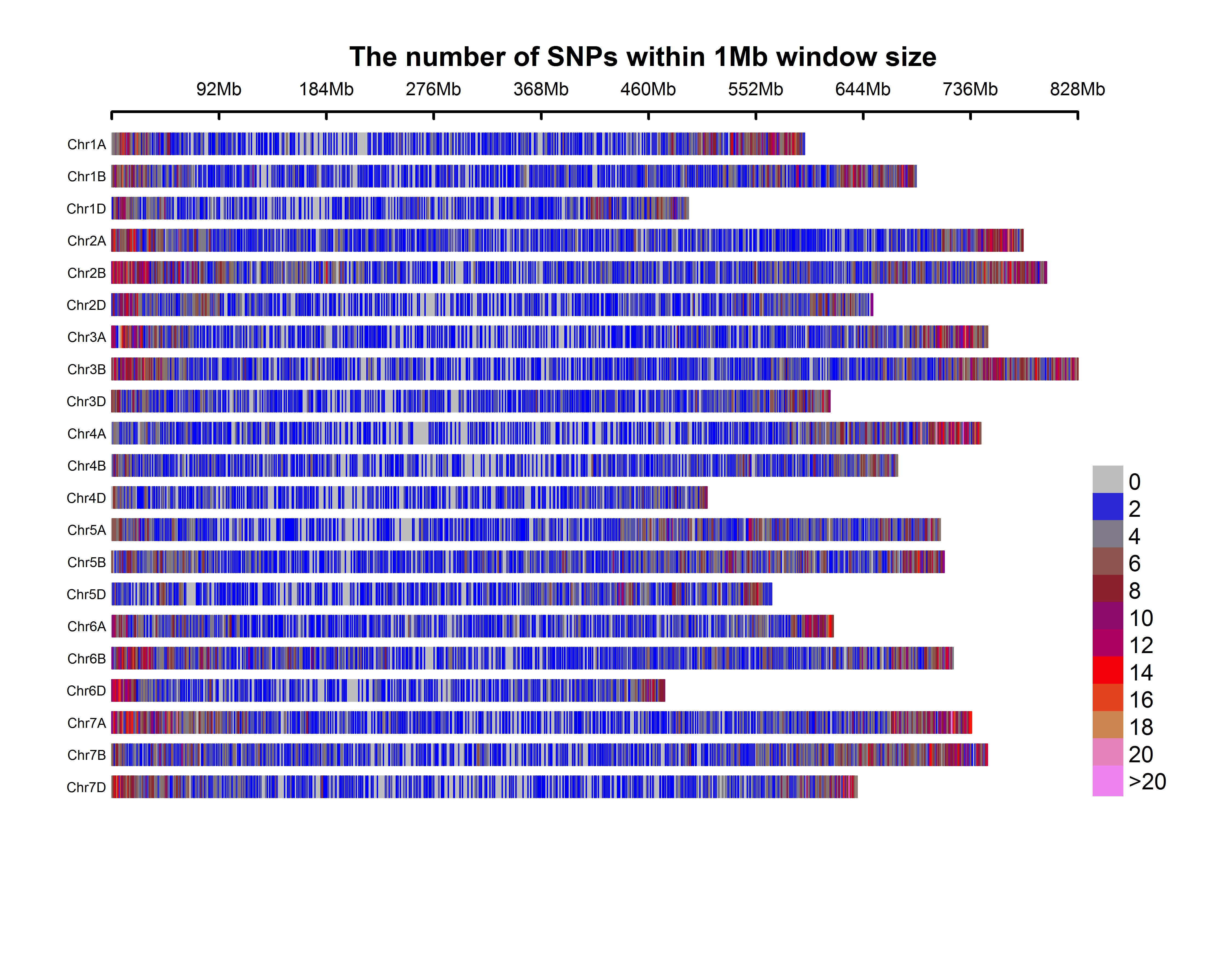

Supplement: Supplementary file 2 — Figure S2. Single nucleotide polymorphism (SNP) distributions on 21 chromosomes in 125 wheat lines, in the vertical axis are the 21 chromosomes. The horizontal axis shows chromosome length (Mb); 0 ~ 20 depicts SNP density (the number of SNPs per window). (JPG 4135 kb) [file 12870_2019_1754_MOESM2_ESM.jpg]

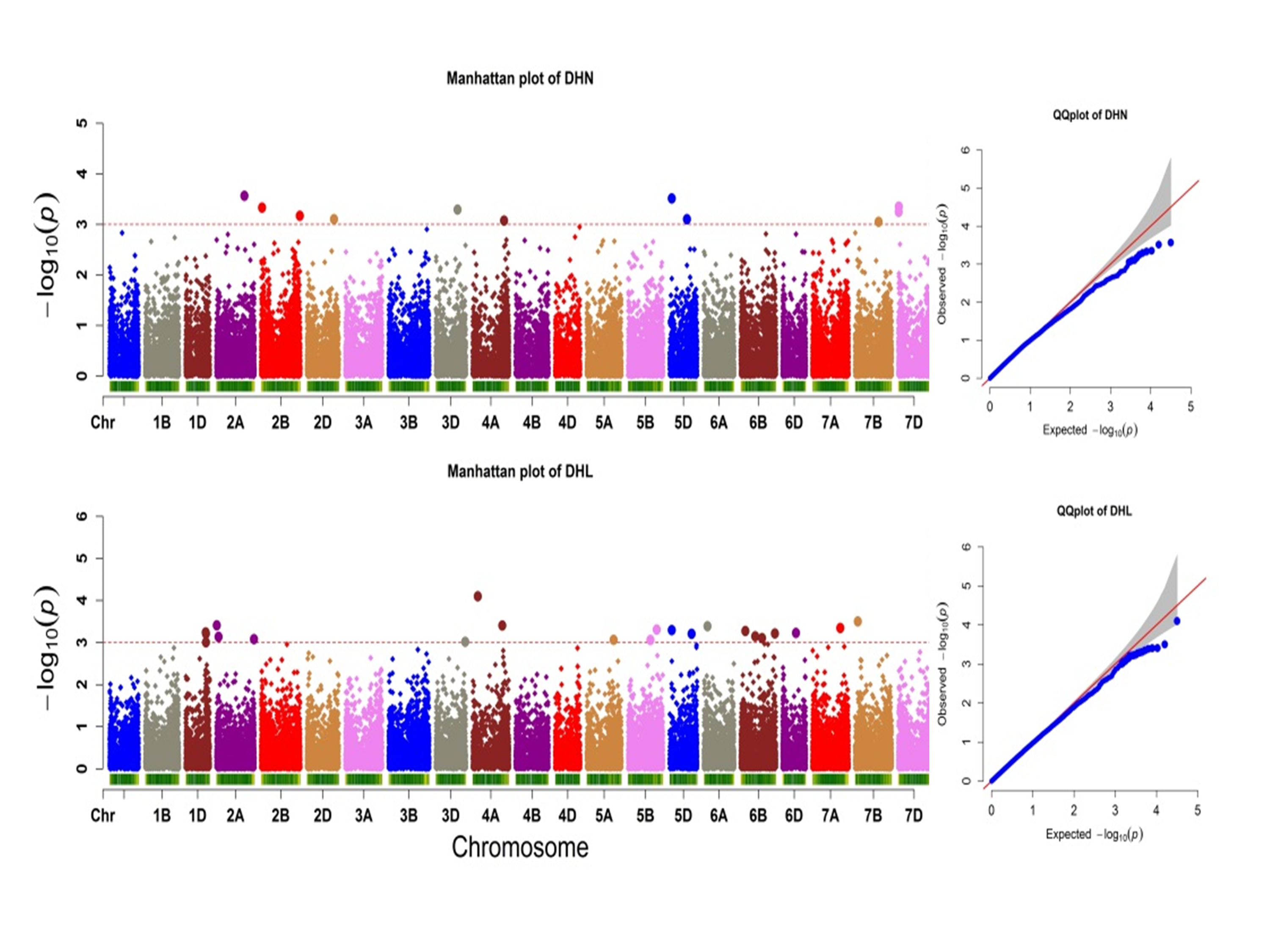

Supplement: Supplementary file 3 — Figure S3-S9. Manhattan plot with QQ plot of days to heading, grain filled duration, plant height, spikes per plant, grain numbers per spike, thousand kernel weight and grain yield under normal (DHN, GFDN, PHN, SPPN, GNSN, TKWN, GYN) and late (DHL, GFDL, PHL, SPPL, GNSL, TKWL, GYL) conditions in 125 wheat lines. (ZIP 38475 kb) [file 12870_2019_1754_MOESM3_ESM.zip › Additional File 3 Figure S3.TIF]

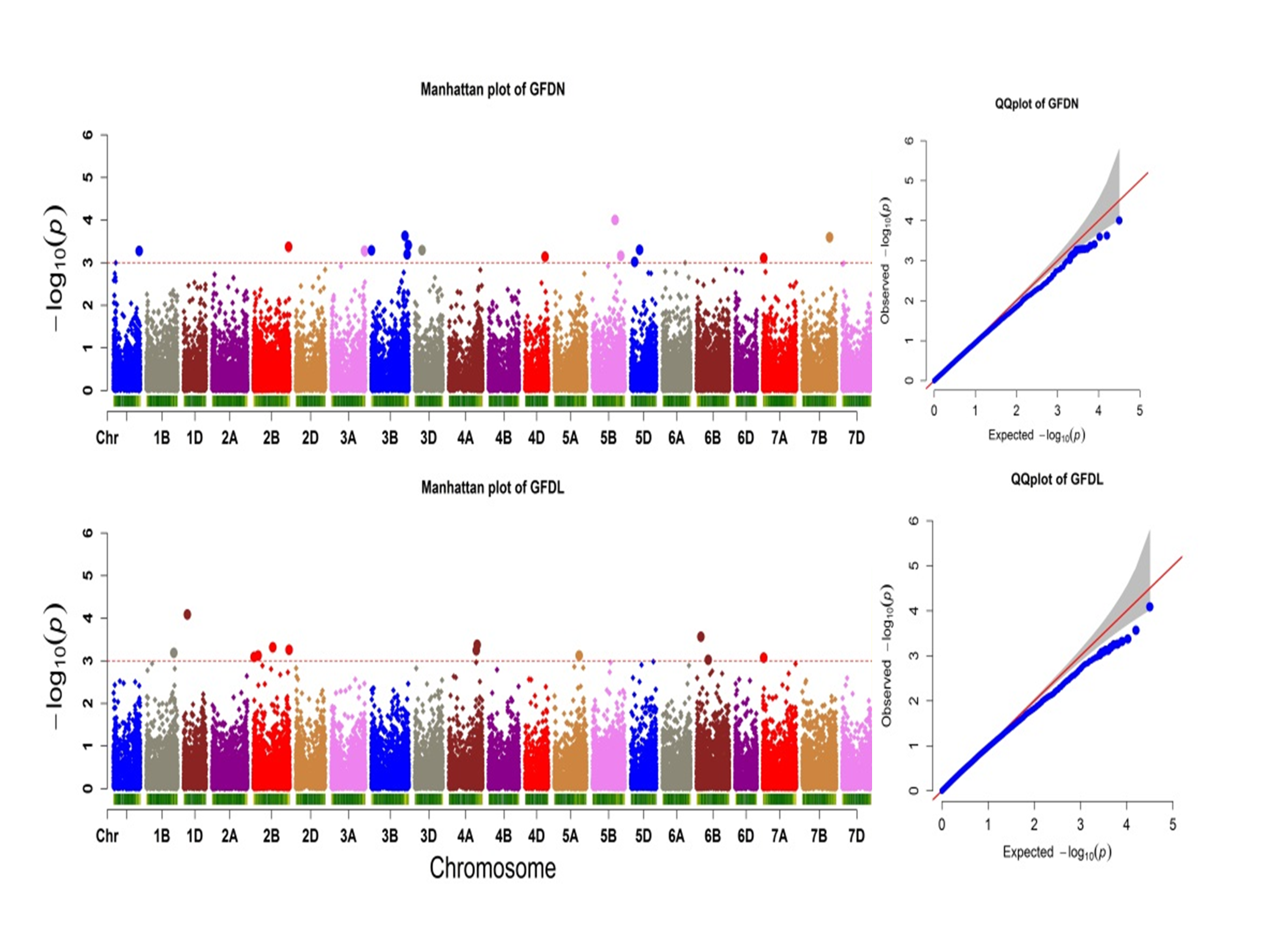

Supplement: Supplementary file 3 — Figure S3-S9. Manhattan plot with QQ plot of days to heading, grain filled duration, plant height, spikes per plant, grain numbers per spike, thousand kernel weight and grain yield under normal (DHN, GFDN, PHN, SPPN, GNSN, TKWN, GYN) and late (DHL, GFDL, PHL, SPPL, GNSL, TKWL, GYL) conditions in 125 wheat lines. (ZIP 38475 kb) [file 12870_2019_1754_MOESM3_ESM.zip › Additional File 4 Figure S4.TIF]

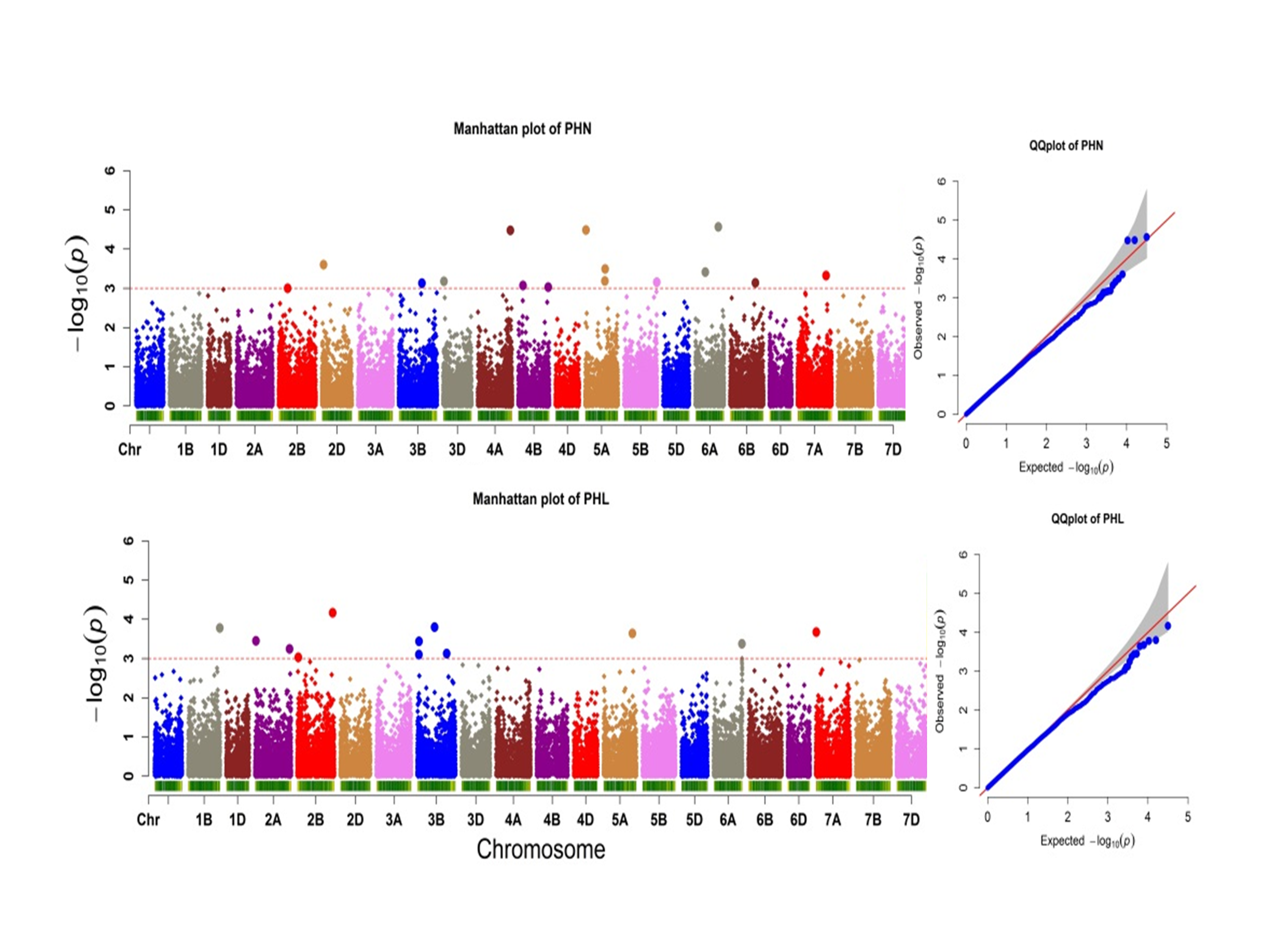

Supplement: Supplementary file 3 — Figure S3-S9. Manhattan plot with QQ plot of days to heading, grain filled duration, plant height, spikes per plant, grain numbers per spike, thousand kernel weight and grain yield under normal (DHN, GFDN, PHN, SPPN, GNSN, TKWN, GYN) and late (DHL, GFDL, PHL, SPPL, GNSL, TKWL, GYL) conditions in 125 wheat lines. (ZIP 38475 kb) [file 12870_2019_1754_MOESM3_ESM.zip › Additional file 5 Figure S5.TIF]

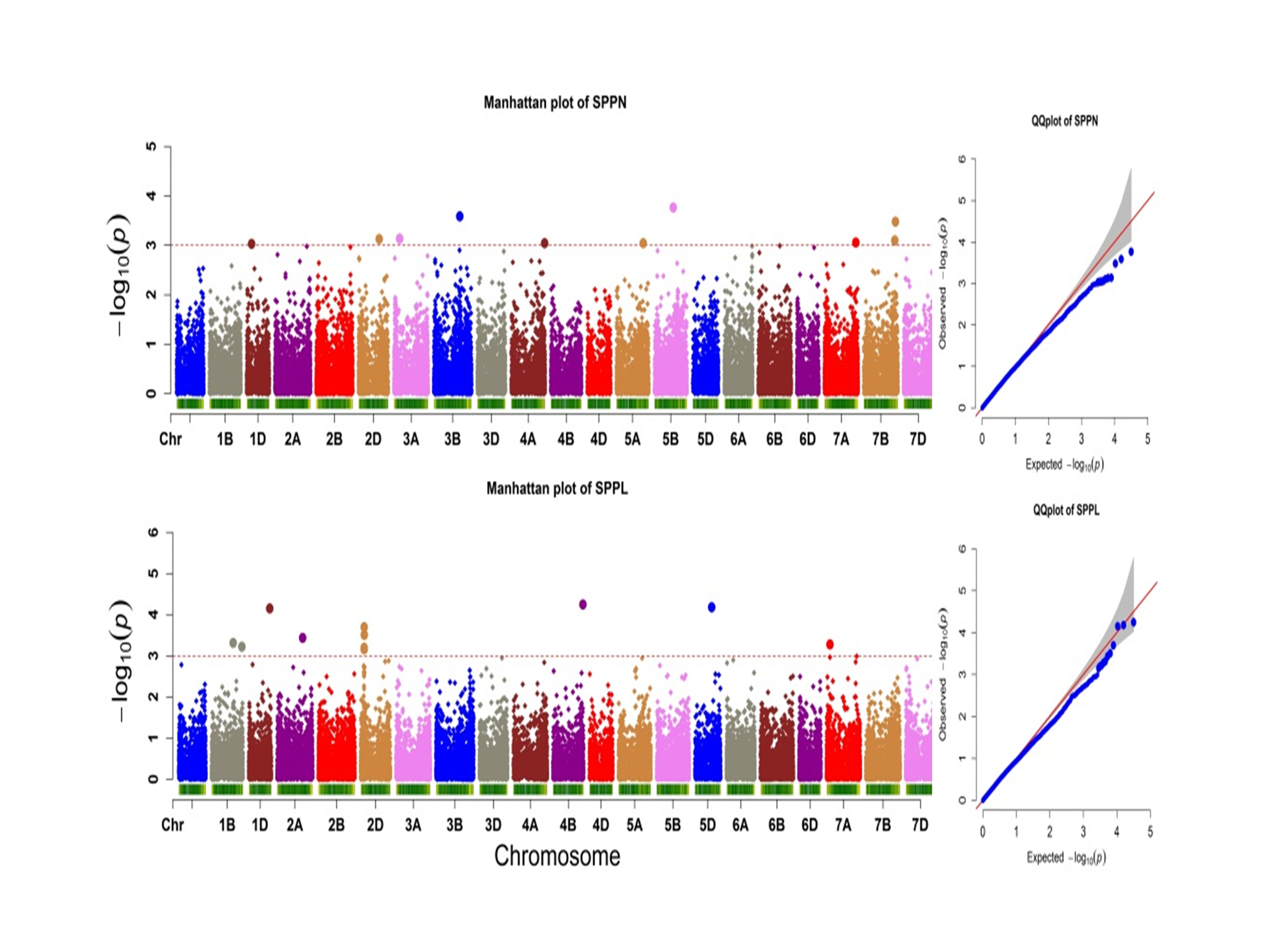

Supplement: Supplementary file 3 — Figure S3-S9. Manhattan plot with QQ plot of days to heading, grain filled duration, plant height, spikes per plant, grain numbers per spike, thousand kernel weight and grain yield under normal (DHN, GFDN, PHN, SPPN, GNSN, TKWN, GYN) and late (DHL, GFDL, PHL, SPPL, GNSL, TKWL, GYL) conditions in 125 wheat lines. (ZIP 38475 kb) [file 12870_2019_1754_MOESM3_ESM.zip › Additional file 6 Figure S6.TIF]

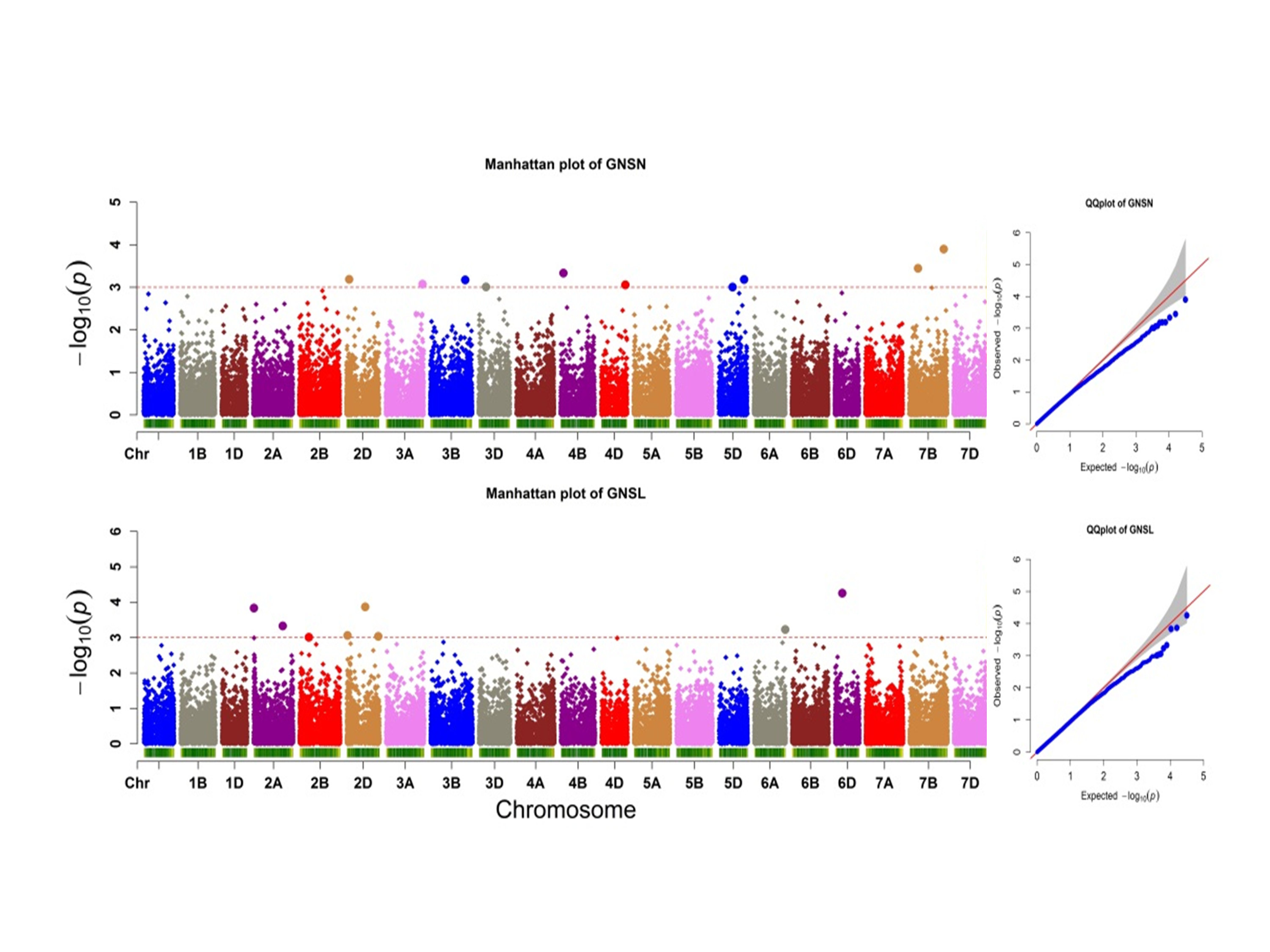

Supplement: Supplementary file 3 — Figure S3-S9. Manhattan plot with QQ plot of days to heading, grain filled duration, plant height, spikes per plant, grain numbers per spike, thousand kernel weight and grain yield under normal (DHN, GFDN, PHN, SPPN, GNSN, TKWN, GYN) and late (DHL, GFDL, PHL, SPPL, GNSL, TKWL, GYL) conditions in 125 wheat lines. (ZIP 38475 kb) [file 12870_2019_1754_MOESM3_ESM.zip › Additional File 7 Figure S7.TIF]

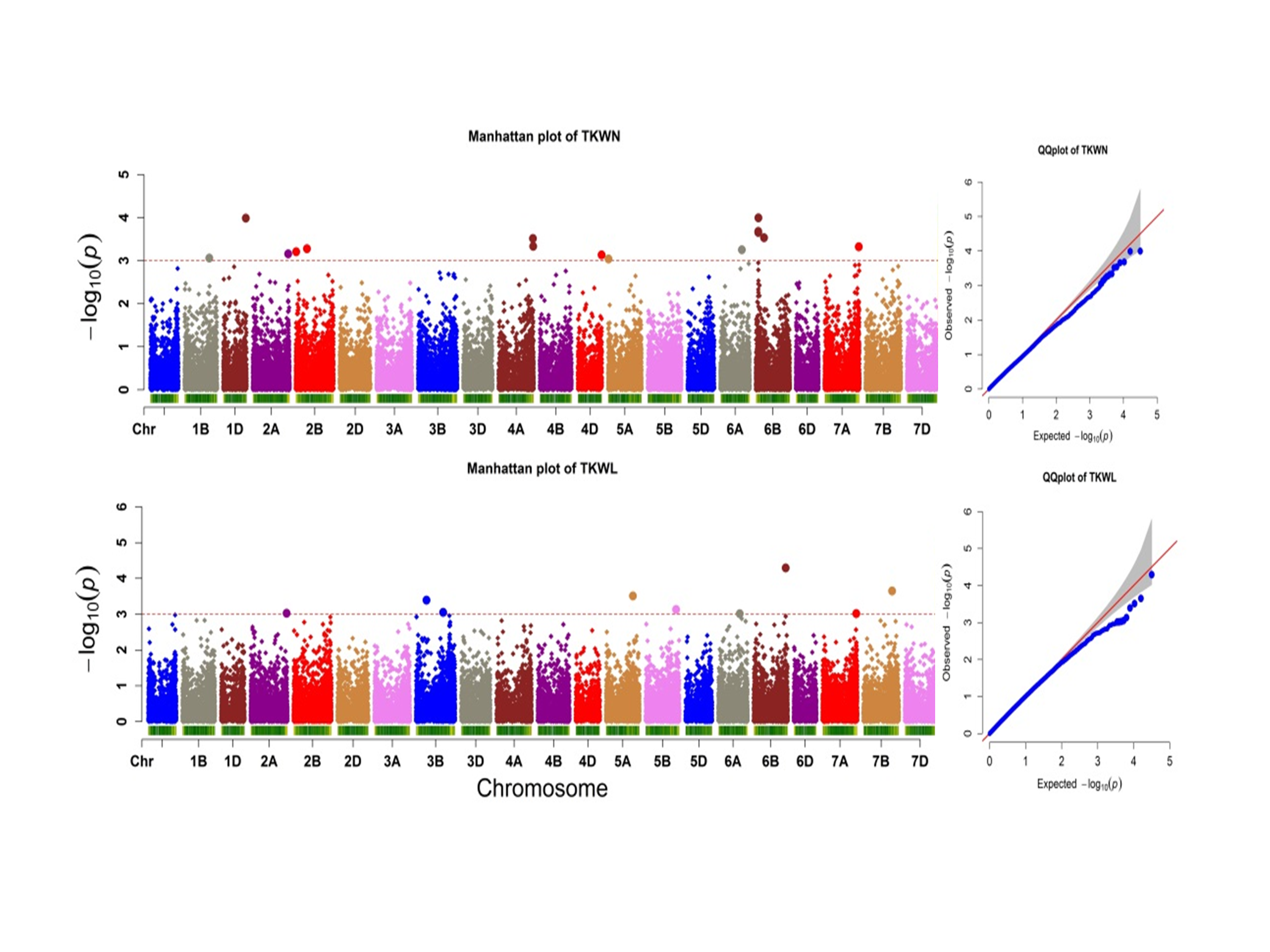

Supplement: Supplementary file 3 — Figure S3-S9. Manhattan plot with QQ plot of days to heading, grain filled duration, plant height, spikes per plant, grain numbers per spike, thousand kernel weight and grain yield under normal (DHN, GFDN, PHN, SPPN, GNSN, TKWN, GYN) and late (DHL, GFDL, PHL, SPPL, GNSL, TKWL, GYL) conditions in 125 wheat lines. (ZIP 38475 kb) [file 12870_2019_1754_MOESM3_ESM.zip › Additional File 8 Figure S8.TIF]

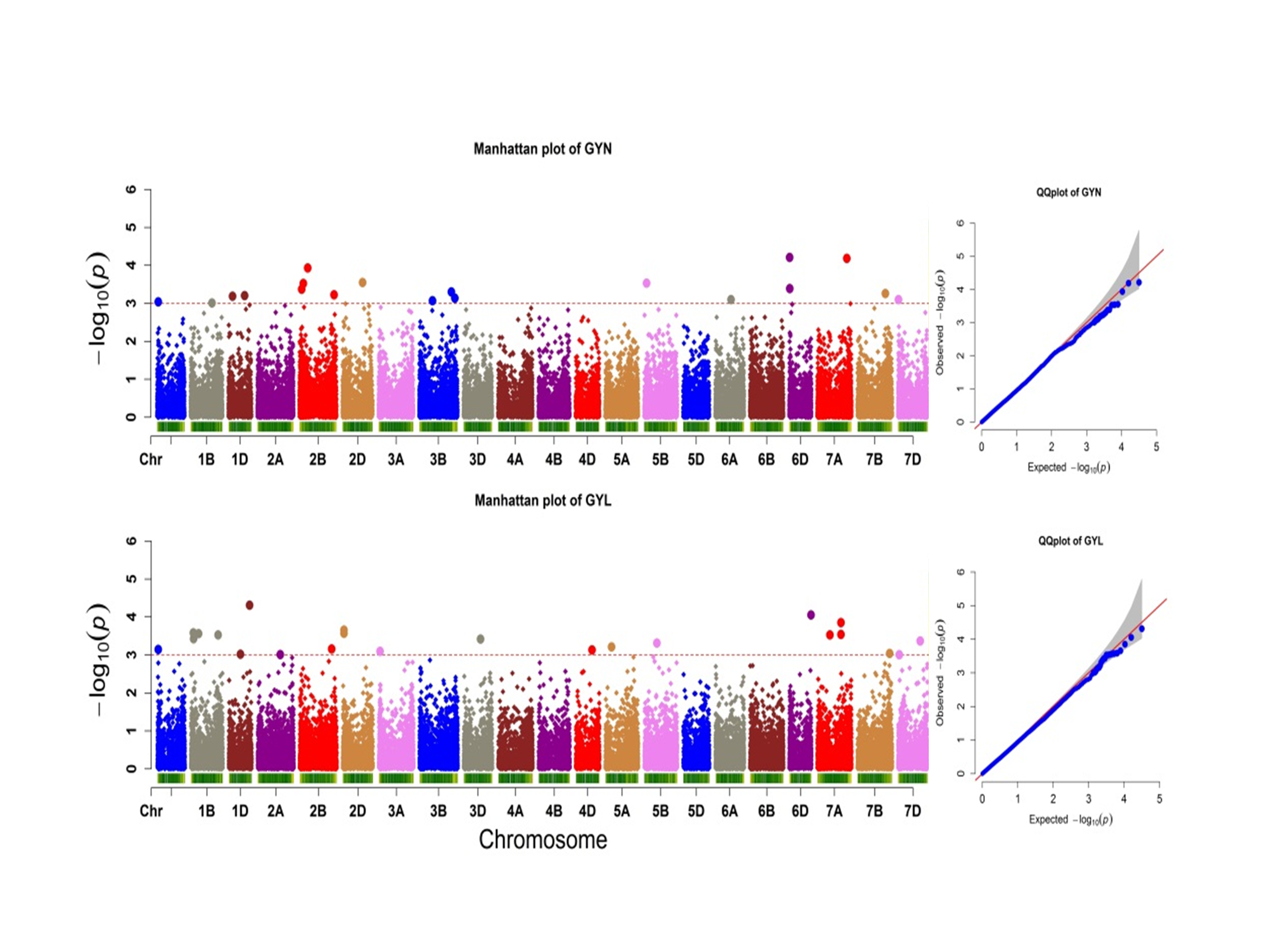

Supplement: Supplementary file 3 — Figure S3-S9. Manhattan plot with QQ plot of days to heading, grain filled duration, plant height, spikes per plant, grain numbers per spike, thousand kernel weight and grain yield under normal (DHN, GFDN, PHN, SPPN, GNSN, TKWN, GYN) and late (DHL, GFDL, PHL, SPPL, GNSL, TKWL, GYL) conditions in 125 wheat lines. (ZIP 38475 kb) [file 12870_2019_1754_MOESM3_ESM.zip › Additional File 9 Figure S9.TIF]

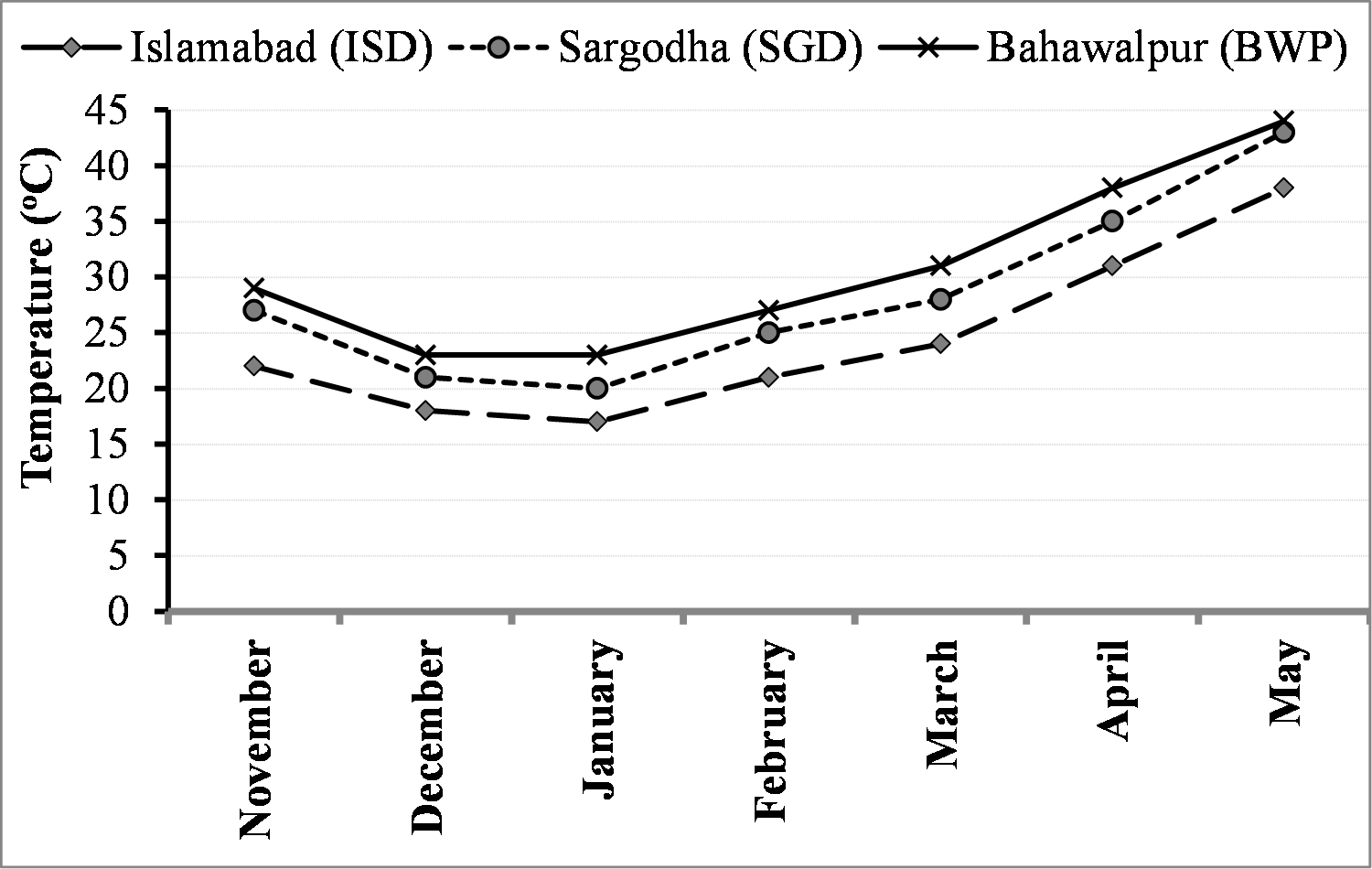

Supplement: Supplementary file 3 — Figure S3-S9. Manhattan plot with QQ plot of days to heading, grain filled duration, plant height, spikes per plant, grain numbers per spike, thousand kernel weight and grain yield under normal (DHN, GFDN, PHN, SPPN, GNSN, TKWN, GYN) and late (DHL, GFDL, PHL, SPPL, GNSL, TKWL, GYL) conditions in 125 wheat lines. (ZIP 38475 kb) [file 12870_2019_1754_MOESM3_ESM.zip › Additional File 10 Figure S10.png]

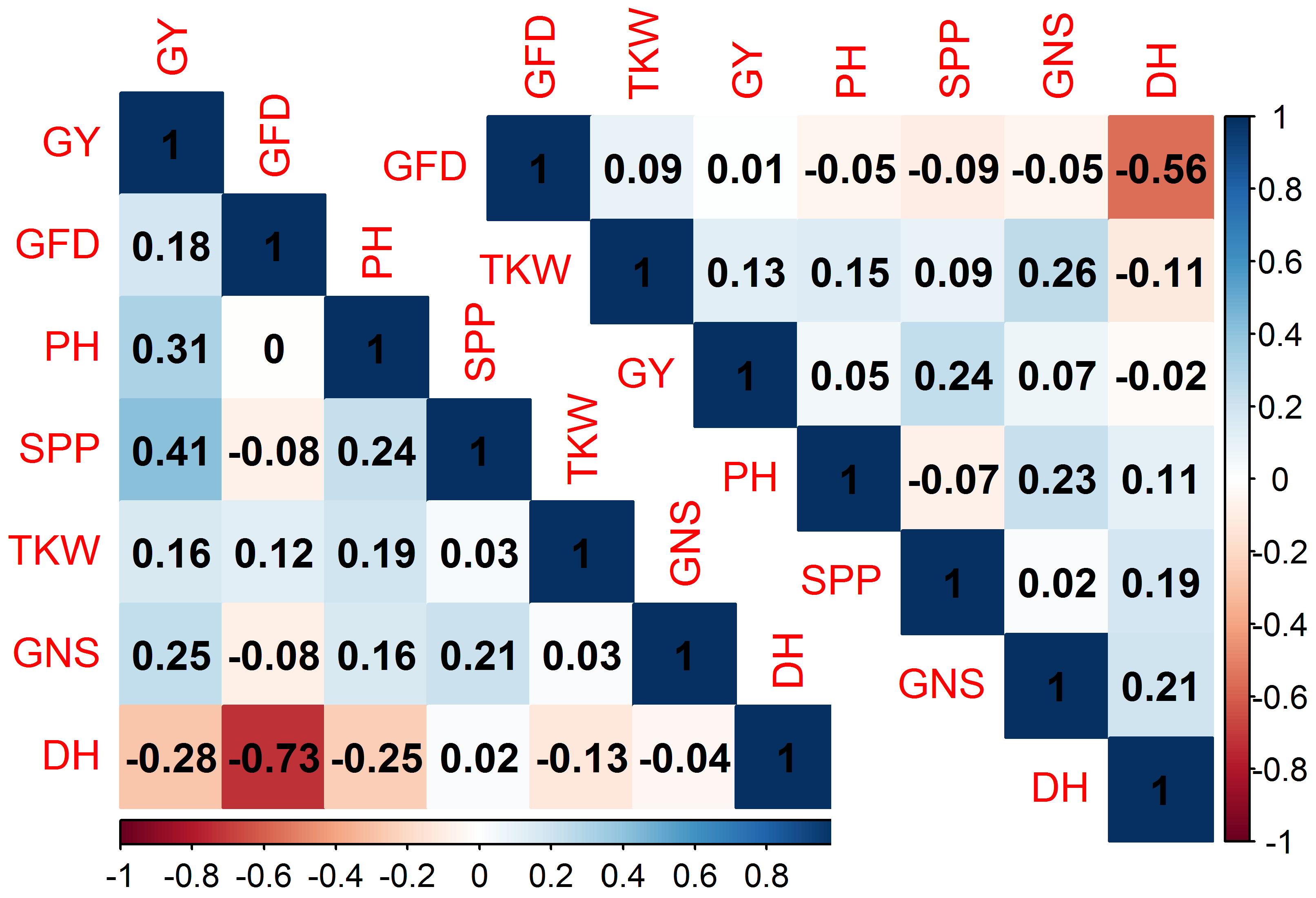

Supplement: Supplementary file 3 — Figure S3-S9. Manhattan plot with QQ plot of days to heading, grain filled duration, plant height, spikes per plant, grain numbers per spike, thousand kernel weight and grain yield under normal (DHN, GFDN, PHN, SPPN, GNSN, TKWN, GYN) and late (DHL, GFDL, PHL, SPPL, GNSL, TKWL, GYL) conditions in 125 wheat lines. (ZIP 38475 kb) [file 12870_2019_1754_MOESM3_ESM.zip › Fig 1.tiff.tif]

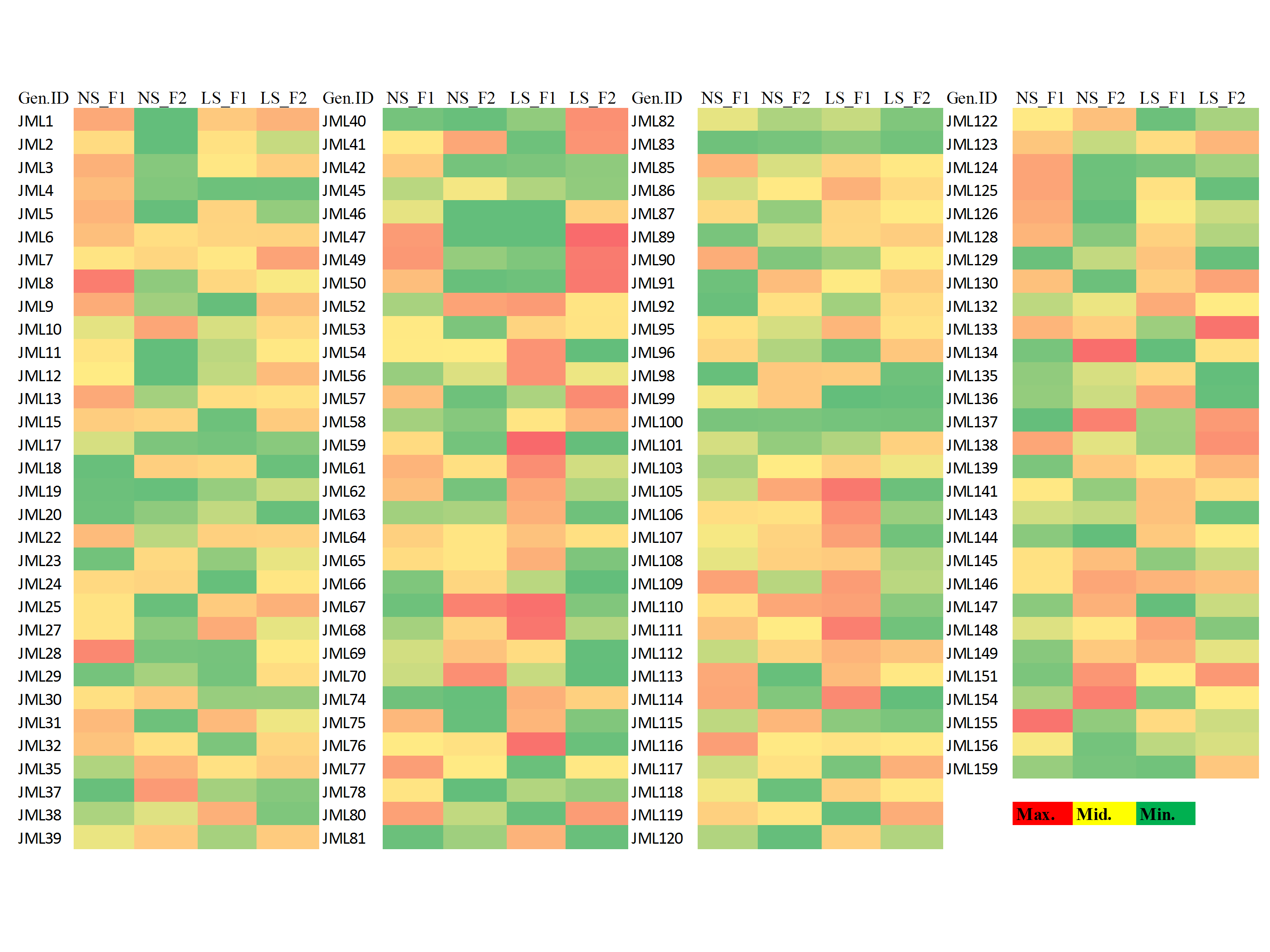

Supplement: Supplementary file 3 — Figure S3-S9. Manhattan plot with QQ plot of days to heading, grain filled duration, plant height, spikes per plant, grain numbers per spike, thousand kernel weight and grain yield under normal (DHN, GFDN, PHN, SPPN, GNSN, TKWN, GYN) and late (DHL, GFDL, PHL, SPPL, GNSL, TKWL, GYL) conditions in 125 wheat lines. (ZIP 38475 kb) [file 12870_2019_1754_MOESM3_ESM.zip › Fig 3.tiff.tif]

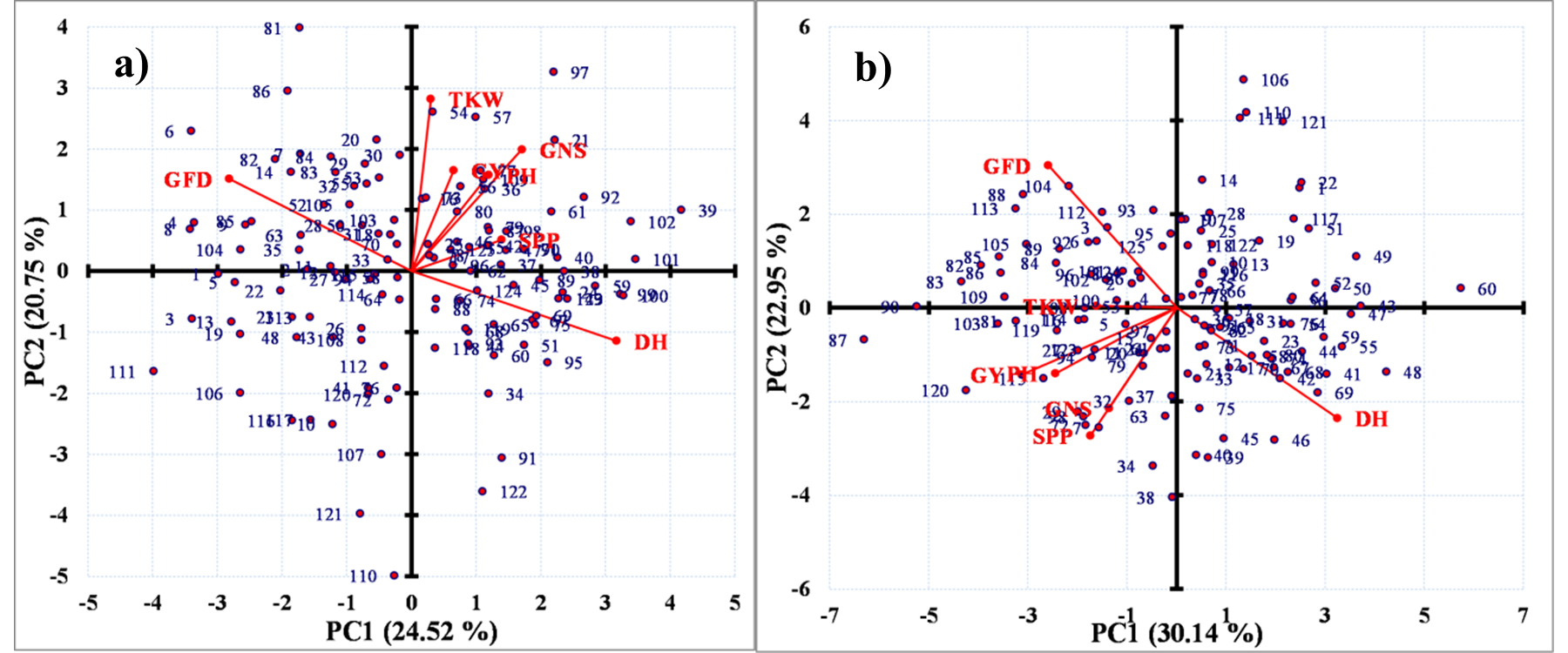

Supplement: Supplementary file 3 — Figure S3-S9. Manhattan plot with QQ plot of days to heading, grain filled duration, plant height, spikes per plant, grain numbers per spike, thousand kernel weight and grain yield under normal (DHN, GFDN, PHN, SPPN, GNSN, TKWN, GYN) and late (DHL, GFDL, PHL, SPPL, GNSL, TKWL, GYL) conditions in 125 wheat lines. (ZIP 38475 kb) [file 12870_2019_1754_MOESM3_ESM.zip › Fig 2.tiff.tif]

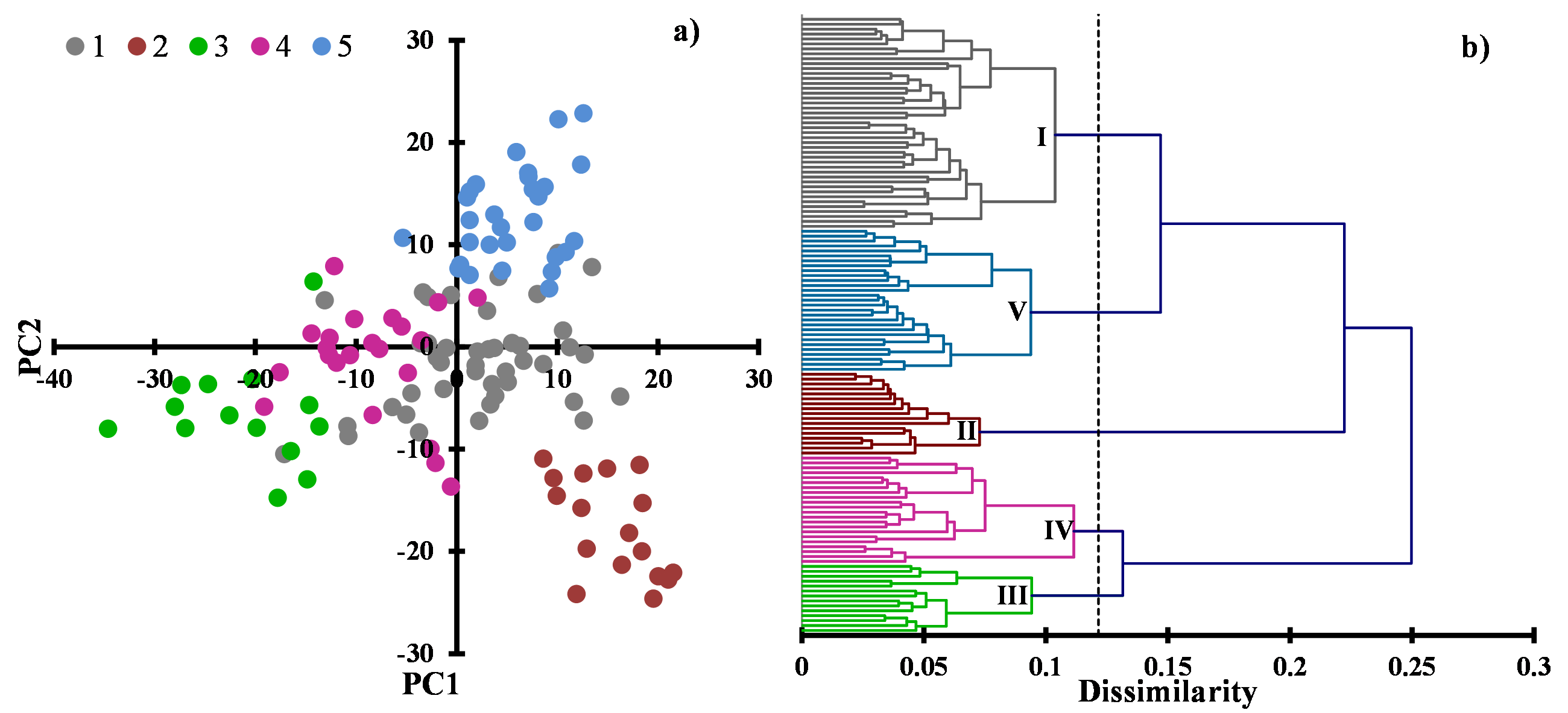

Supplement: Supplementary file 3 — Figure S3-S9. Manhattan plot with QQ plot of days to heading, grain filled duration, plant height, spikes per plant, grain numbers per spike, thousand kernel weight and grain yield under normal (DHN, GFDN, PHN, SPPN, GNSN, TKWN, GYN) and late (DHL, GFDL, PHL, SPPL, GNSL, TKWL, GYL) conditions in 125 wheat lines. (ZIP 38475 kb) [file 12870_2019_1754_MOESM3_ESM.zip › Fig 4.tif.tif]

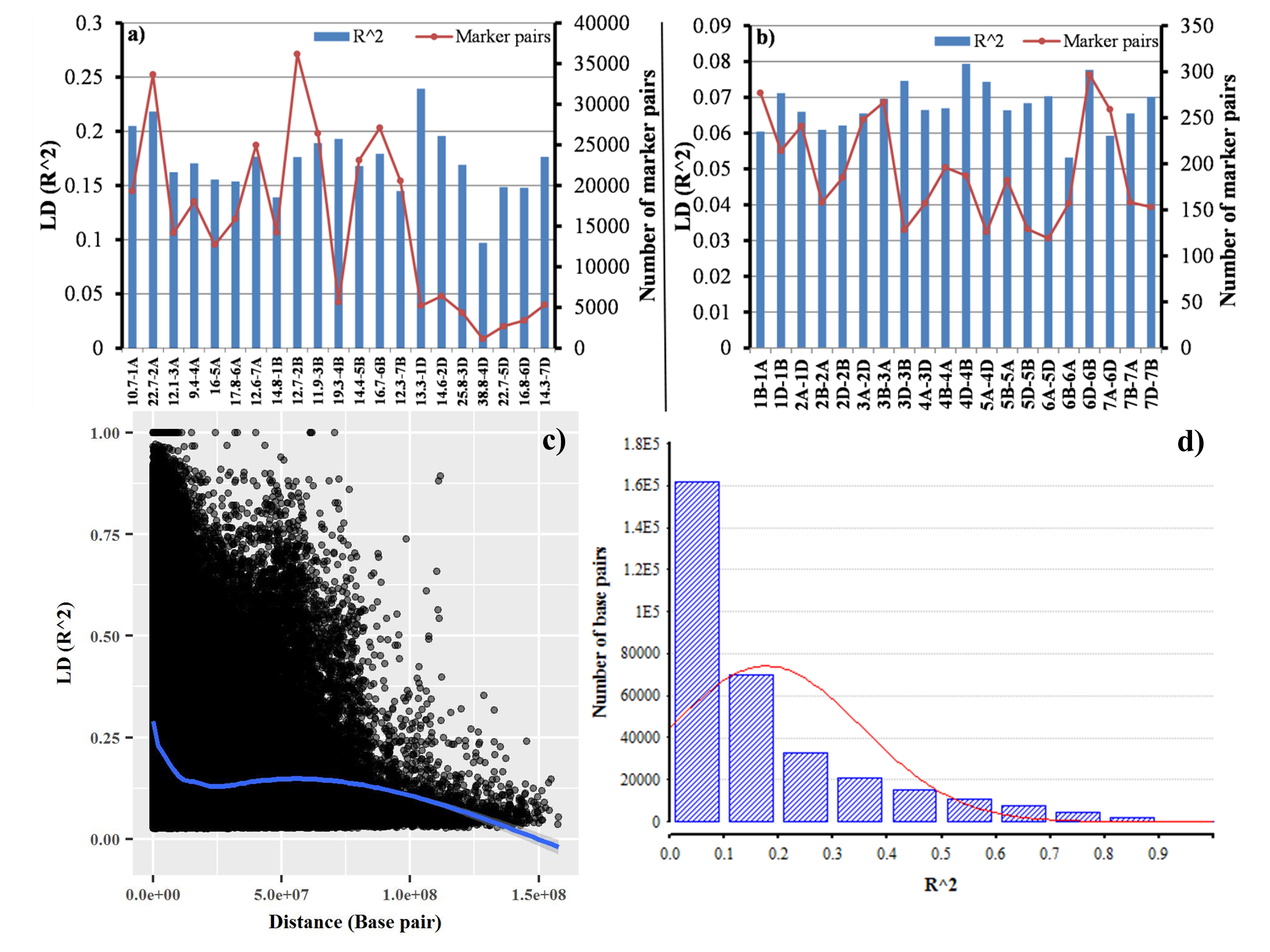

Supplement: Supplementary file 3 — Figure S3-S9. Manhattan plot with QQ plot of days to heading, grain filled duration, plant height, spikes per plant, grain numbers per spike, thousand kernel weight and grain yield under normal (DHN, GFDN, PHN, SPPN, GNSN, TKWN, GYN) and late (DHL, GFDL, PHL, SPPL, GNSL, TKWL, GYL) conditions in 125 wheat lines. (ZIP 38475 kb) [file 12870_2019_1754_MOESM3_ESM.zip › Fig 5.tiff.tif]

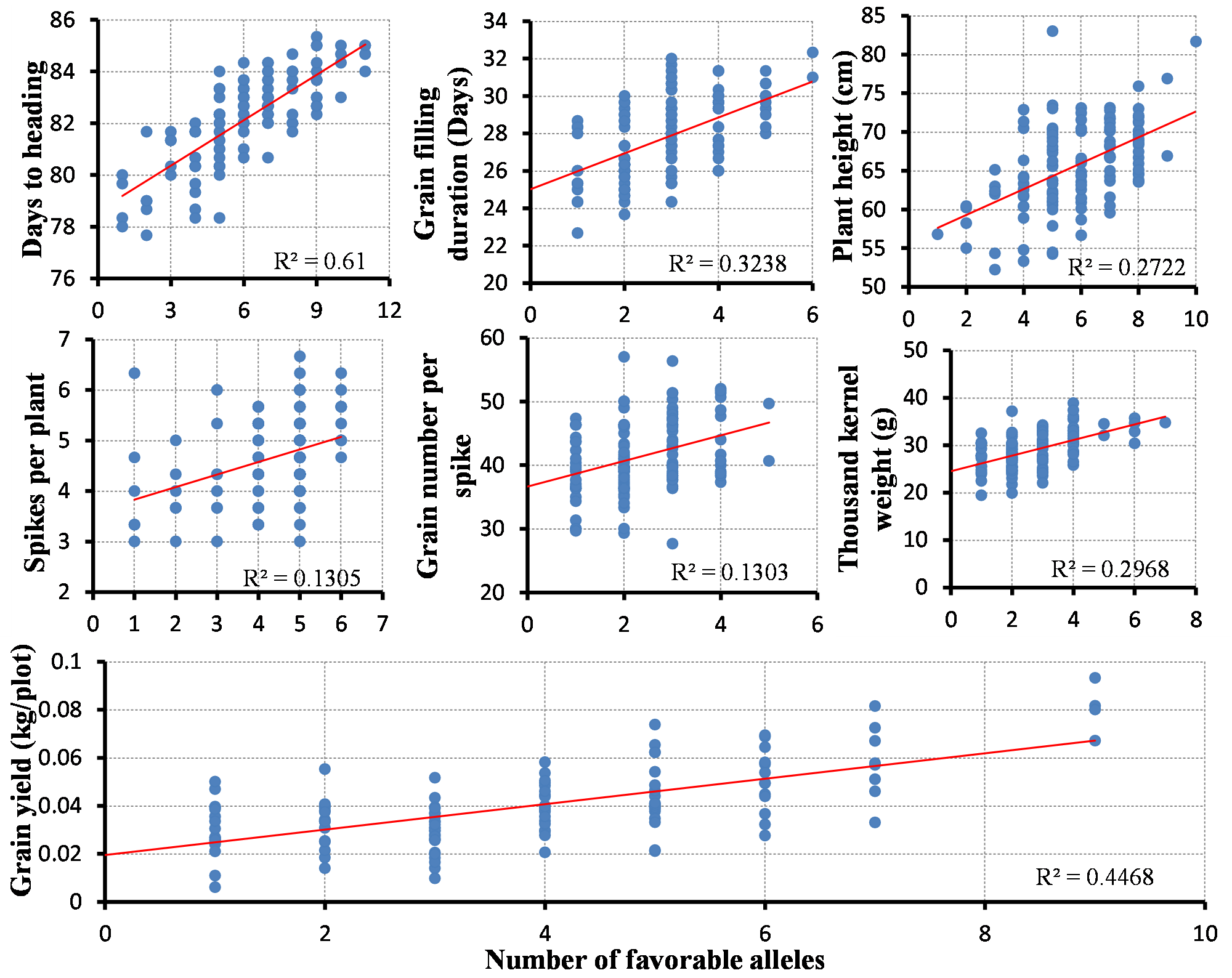

Supplement: Supplementary file 3 — Figure S3-S9. Manhattan plot with QQ plot of days to heading, grain filled duration, plant height, spikes per plant, grain numbers per spike, thousand kernel weight and grain yield under normal (DHN, GFDN, PHN, SPPN, GNSN, TKWN, GYN) and late (DHL, GFDL, PHL, SPPL, GNSL, TKWL, GYL) conditions in 125 wheat lines. (ZIP 38475 kb) [file 12870_2019_1754_MOESM3_ESM.zip › Fig 7.tiff.tif]

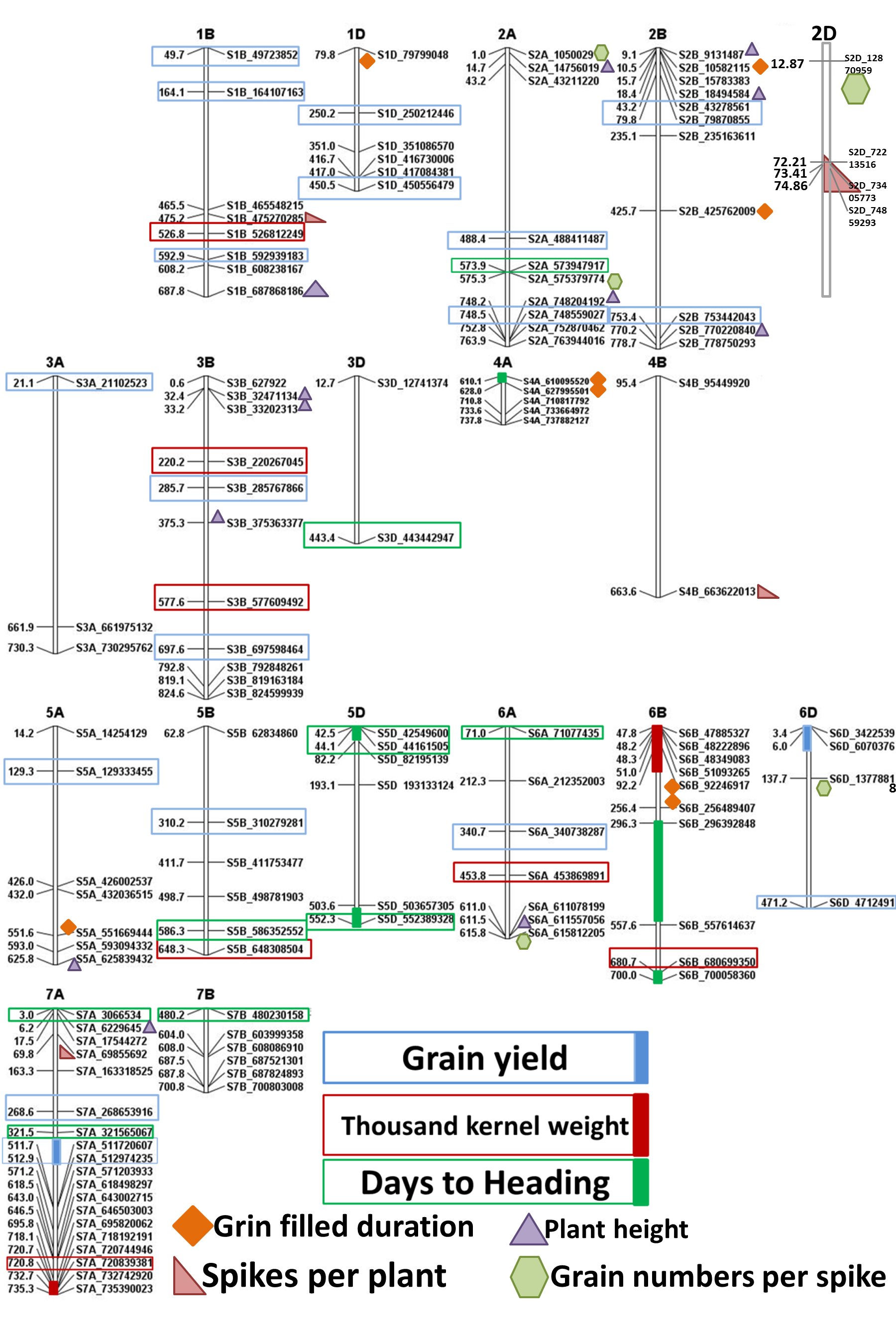

Supplement: Supplementary file 3 — Figure S3-S9. Manhattan plot with QQ plot of days to heading, grain filled duration, plant height, spikes per plant, grain numbers per spike, thousand kernel weight and grain yield under normal (DHN, GFDN, PHN, SPPN, GNSN, TKWN, GYN) and late (DHL, GFDL, PHL, SPPL, GNSL, TKWL, GYL) conditions in 125 wheat lines. (ZIP 38475 kb) [file 12870_2019_1754_MOESM3_ESM.zip › Fig 6.Tiff..tif]
