# Supplementary material for: Genome-wide association studies of seven agronomic traits under two sowing conditions in bread wheat
Source: BMC Plant Biol. 2019 Apr 17;19:149. doi: 10.1186/s12870-019-1754-6 (PMC6475106; doi:10.1186/s12870-019-1754-6)
Supplement: Supplementary file 3 — Figure S3-S9. Manhattan plot with QQ plot of days to heading, grain filled duration, plant height, spikes per plant, grain numbers per spike, thousand kernel weight and grain yield under normal (DHN, GFDN, PHN, SPPN, GNSN, TKWN, GYN) and late (DHL, GFDL, PHL, SPPL, GNSL, TKWL, GYL) conditions in 125 wheat lines. (ZIP 38475 kb) [file 12870_2019_1754_MOESM3_ESM.zip › Jamil 20.3.19.docx]

**Genome-wide association studies of seven agronomic traits under two sowing conditions in bread wheat**

Muhammad Jamil^1*^, Aamir Ali^1^, Alvina Gul^2^, Abdul Ghafoor^3^, Abdul Aziz Napar^4^, Amir M.H. Ibrahim^5^, Naima Huma Naveed^1^, Nasim Ahmad Yasin^6^, Abdul Mujeeb-Kazi^7^

^1^Department of Botany, University of Sargodha, Sargodha, Punjab, Pakistan ^1^*([jshahid80@yahoo.com](mailto:jshahid80@yahoo.com)), ^1^([draamirali73@gmail.com](mailto:draamirali73@gmail.com)), ^1^([naimahuma@yahoo.com](mailto:naimahuma@yahoo.com))

^2^Atta-ur-Rehman School of Applied Biosciences (ASAB), National University of Science and Technology (NUST), Islamabad, Pakistan ([Alvina_gul@yahoo.com](mailto:Alvina_gul@yahoo.com))

^3^Plant Genetic Resources Institute (PGRI), National Agriculture Research Center (NARC), Islamabad, Pakistan ([ghafoor59pk@yahoo.com](mailto:ghafoor59pk@yahoo.com))

^4^Institute of Plant Sciences, University of Sind Jamshoro, Sind, Pakistan ([abdulaziznapar@yahoo.com](mailto:abdulaziznapar@yahoo.com))

^5^Soil and Crop Sciences Department, Texas A&M University, College Station, USA ([aibrahim@tamu.edu](mailto:aibrahim@tamu.edu))

^6^Horticulture wing, University of the Punjab, Pakistan [(nasimhort@yahoo.com](mailto:(nasimhort@yahoo.com))

^7^Texas A&M Agrilife Research & Extension Center, Amarillo, Texas, USA ([kayshtr@gmail.com](mailto:kayshtr@gmail.com))

* Corresponding author: e-mail: [jshahid80@yahoo.com](mailto:jshahid80@yahoo.com) ; Cell phone: 0092-333-6291799

**Abstract**

**Background**

Wheat is a cool seasoned crop requiring low temperature during grain filling duration and therefore increased temperature causes significant yield reduction. A set of 125 spring wheat genotypes from International Maize and Wheat Improvement Centre (CIMMYT-Mexico) was evaluated for phenological and yield related traits at three locations in Pakistan under normal sowing time and late sowing time for expose to prolonged high temperature. With the help of genome-wide association study using genotyping-by-sequencing, marker trait associations (MTAs) were observed separately for the traits under normal and late sown conditions.

**Results**

Significant reduction ranging from 9% to 74% was observed in all traits under high temperature. Especially 30%, 25%, 41% and 66% reduction was observed for days to heading (DH), plant height (PH), Spikes per plant (SPP) and yield respectively. We identified 55954 single nucleotide polymorphisms (SNPs) using genotyping by sequencing of these 125 hexaploid spring wheat genotypes and conducted genome-wide association studies (GWAS) for days to heading (DH), grain filled duration (GFD), plant height (PH), spikes per plant (SPP), grain number per spike (GNS), thousand kernel weight (TKW) and grain yield per plot (GY). Genomic regions identified through GWAS explained up to 13% of the phenotypic variance, on average. A total of 139 marker-trait associations (MTAs) across three wheat genomes (56 on chromosome A, 55 on B and 28 on D) were identified for all the seven traits studied. For days to heading, 20; grain filling duration, 21; plant height, 23; spike per plant, 13; grain numbers per spike, 8; thousand kernel weight, 21 and for grain yield, 33 MTAs were detected under normal and late sown conditions.

**Conclusions**

This study identifies the essential resource of genetics research and underpins the chromosomal regions of seven agronomic traits under normal and high temperature. Significant relationship was observed between the number of favored alleles and trait observations. Fourteen protein coding genes with their respective annotations have been searched with the sequence of seven MTAs which were identified in this study. These findings will be helpful in the development of a breeder friendly platform for the selection of high yielding wheat lines at high temperature areas.

**Keywords:** Wheat, Heat tolerance, Genotyping-by-sequencing, SNPs, GWAS

**Background**

As the world population will be increased to 9 billion at the end of 21^st^ century, it is predicted that the food demand, especially for wheat, will be increased by 50% by 2030 and 70% by 2050 [1-2]. On the other hand, the mean temperature in South Asia will increase by 4 ^o^C until 2050 or by end of this century [3]. Significant wheat yield losses of 32% to 39% worldwide [4] and 40 % in less developed-irrigated wheat growing areas [5] have been reported. During grain filling duration, high temperature (>30 ^o^C) also occurs in 40% of the temperate zones that grow 36 million ha of wheat [6].

Around 6% decline in global wheat production [7] and 3-4 % in indo-gigantic planes [8] has been assessed for each degree rise in temperature during the reproductive stage of the crop. High temperature decreases chlorophyll content and photosynthetic capacity of leaves [9], decreases the grain number per spike due to floret abortion, and lowers the grain weight [10]. Grain filling rate and ultimately the yield are highly affected as the temperature increases above 30^o^C during anthesis [11]. Stone and Nicolas [12] reported that exposure of 32 ^o^C for four days reduced yield by 23%. Losses due to heat stress are phenomenal and compelling wheat researchers to focus more on heat stress as compared to other abiotic stresses such as drought, metal or radiation etc.

To cope with high temperature in wheat during reproductive stage is desired by the breeders. Traits affected by high temperature are investigated to develop heat tolerant wheat cultivars. It is therefore essential to grow wheat lines under normal and heat stressed conditions to examine the variability. Using conventional wheat breeding approaches, valuable traits related to stress tolerance have been incorporated. However, it is time consuming due to limited field screening methods and laborious due to low probability of combining coveted alleles [13]. Progeny screening can be more precise and genetic gains can be accelerated if the conventional approaches are aided by molecular based techniques.

To dissect the genetic basis of the quantitative traits associated with heat tolerance, loci for grain filling duration and heat tolerance were mapped [14-15] with few simple sequence repeats (SSRs). Other quantitative trait loci (QTL) for heat tolerance using SSRs were then identified [16-17]. These studies were based on low marker density using SSRs. QTL mapping identifies genomic regions with low resolution; it hinders their use in diverse germplasm [18].

In order to obtain better genomic dissection of complex quantitative traits like heat tolerance, a high-throughput and robust genotyping approach is in demand [19]. Most recently Diversity Arrays Technology (DArT) markers were used to map agronomic traits in heat prone areas [20]. Genotyping with SSRs and DArT is cost-ineffective, laborious and less accuracy of prediction [19]. Genotyping-by-sequencing (GBS) is a high-throughput, cost effective, next generation sequence-based technology [21] which detects single nucleotide polymorphisms (SNP). GBS markers allow researchers to obtain high prediction accuracy as compared to previously used array of hybridization-based markers [22].

Based on such high density SNP markers, genome-wide association study (GWAS) can inspect large gene pools representative of diverse breeding reservoirs. GWAS is the most suitable approach to locate robust QTLs that show effect in both normal and stressed conditions. Recently GWAS has been effectively used to map QTLs for grain weight, drought stress, floret fertility, grain architecture and starch granule size [23-27].

Our objective was to use a high-density (GBS) platform through GWAS to identify chromosomal regions potentially associated with days to heading (DH), grain filling duration (GFD), plant height (PH), spikes per plant (SPP), grain number per spike (GNS), thousand kernel weight (TKW) and grain yield (GY) under normal and late sown conditions. Information from this scientific work will help wheat breeders for genetic improvement in the development of heat tolerant lines.

**Results**

**Phenotypic assessment**

Description of seven studied trait at three locations under two treatments (NS: Normal and LS: Late sown) indicates significant (p-value <0.01) effect on 125 genotypes (Table 1). For each trait, the difference recorded between NS and LS condition across all the environments was above the critical difference *i.e.* least significant difference (LSD at p-value 0.05). It was confirmed that the late sown trials faced high temperature from anthesis to physiological maturity that affects each studied trait. Yield losses due to high temperature ranged from 66% to 76% on average at three locations. Plant height was reduced by 25, 30 and 20% on average at Islamabad (ISD), Sargodha (SGD) and Bahawalpur (BWP) respectively. Average days to heading were decreased from 126 to 88 at ISD, 123 to 81 at SGD, 108 to 78 at BWP. Grain filling duration was decreased by 9 to 34 percent. Mean square of genotypes was also separately calculated for each location and treatment as a one-way source of variation to observe the inherent potential of the lines under study. Mean square of genotypes, locations and treatments for all studied traits were found to be significant (p-value <0.001) with the degree of precision (CV%) ranging from 6.5 to 18.52 (Table 2). Interaction mean squares for all the traits except GFD were significant.

In NS conditions, GY was positively correlated with SPP (r = 0.24) and GNS was correlated with TKW (r = 0.26), DH (r = 0.21) and PH (r = 0.23); whereas GFD was significantly negative correlated with DH (r = -0.56) and positive with SPP (r = 0.20) at p-value < 0.05 (Fig 1). In LS conditions, GY was significantly negative correlated with DH (r = - 0.28) and positively correlated with GFD (r=0.18), PH (r= 0.31), SPP (0.41) and GNS (r=0.25); whereas DH was significantly negative correlated with GFD (r= - 0.73) and PH (r= - 0.25) at p-value < 0.05.

Biplots were constructed for NS and LS conditions separately. The two principal components explained 45.27% variability in NS and 53.09% variability in LS conditions. Traits TKW, GNS, PH, GY, SPP occupied the same section in the biplot which indicates similar pattern of variability among these traits. These traits were found correlated to each other. On the other hand GFD and DH were opposite to each other in two dimensional biplot spread indicates their highly negative correlation. Genotypes with serial number 6, 81, 86, 97 and 39 performed well in normal sown conditions while 111, 120, 121, 110 performed poorly under NS conditions. Under LS conditions, genotypes with serial numbers 87, 120, 38 and 34 were found efficient with respect to GY, GNS, PH and SPP traits (Fig 2). Genotype with serial number 34 was (waxwing*2/Heilo), the released wheat variety “Lemu”, genotype with serial number 38 was developed from Kachu#1/Kiritati//Kachu while the genotype with serial number 87 has well known synthetic wheat in its pedigree. Genotype with serial number 120 was the line having Pastor, Kakuna, Milan, Kauz in its pedigree, further detail has been given in additional file 1: Table S1. Genotypes with serial numbers 104, 88 and 113 showed the lowest DH and the highest GFD under LS condition.

To check the overall performance of each genotype in NS and LS conditions, squared cosine values were calculated from principal component analysis (PCA). In Fig 3, red colored rectangles indicate significant squared cosines of genotypes at F1 (PC1) and F2 (PC2). Genotypes with significant scores in LS conditions performed best with respect to all seven traits. These genotypes can be the genetic resource in the development of heat tolerant cultivars.

**Marker distribution, Principal component analysis (PCA), Agglomerative hierarchical clustering (AHC) and Linkage disequilibrium (LD)**

In total 87096 SNPs were identified but 55,954 GBS markers were filtered with MAF >0.05, average polymorphism information content (PIC) and diversity was 0.28 and 0.32, respectively. Out of these 55,954 markers, 35% were from A genome, 41% from B genome and 24% from D (Table 3). Chromosome 2B has the highest number of markers (4,248), while 4D has the lowest (1180). Average physical distance between two adjacent SNP loci (SNP-density) along each chromosomal length can be found in additional file 2: Figure S2.

Five sub-groups (Fig 4a) were expressed by 125 genotypes after the distribution along two principal components (PCs) and this finding was further validated through AHC analysis which confirmed five distinct clusters. Sub-group 3, 5 and 2 were distinctly spread in two dimensional space (Fig 4a). Range of dissimilarity index was 0.05 to 0.25 which elucidated five clusters at cutoff value of 0.12 (Fig 4b). Between-class variance decomposition for the optimal classification was 0.022 (15.51%). Range of distance between each class centroid was 0.178 to 0.406. Clusters 1 to 5 contained 43, 17, 14, 22 and 29 genotypes respectively. Each genotype with its corresponding score of five PCs and its designated cluster can be seen in additional file 1: Table S1.

Linkage disequilibrium was calculated from 3154450 pairs using 100 marker sliding window operation out of which 16% of the pairs were with zero LD and 12% of the pairs were found in significant (p-value <0.05) LD. Out of these significant pairs, inter-and intra-chromosomal LD was calculated for 3838 and 320510 pairs respectively. Out of 320510, there were 162362 (51%) intra-chromosomal pairs which showed > 0.1 LD with an average of 0.293 within 16.70 mega base pair (Mb) throughout the whole genome. There were 504 inter-chromosomal pairs with >0.1 r^2^ LD with an average of 0.151. Intra-chromosomal LD ranged from 0.1 at chromosome 4D within 38.84 Mb pair distance to 0.24 r^2^ at chromosome 1D within 13.33Mb pair distance (Fig 5a). Average LD on A, B and D-genome was 0.177, 0.17 and 0.168 with in the average 14.62, 14.58 and 20.19 Mb pairs distance respectively. Inter-chromosomal LD was noted between 0.06 to 0.08 r^2^ (Fig 5b). The extent of (threshold LD >0.1 R^2^) LD-decay was observed at 25Mb pair distance (Fig 5c). A total of 162866 pairs were identified having >0.1 r^2^ LD (Fig 5d).

**Marker-trait associations (MTAs)**

A total of 139 MTAs were identified out of which 69 were identified in NS experiments and 70 were found under LS conditions. Among them, 56, 55 and 28 were on genome A, B and D, respectively (Table 4). Marker S4A_610095520 was found to be associated with two (DH, GFD) traits in LS conditions; S7B_700803008 was linked with SPP in NS and GY in LS conditions and marker S6A_453869891 was identified for TKW in both NS and LS conditions (Table 4).

Six genomic regions were identified in NS and 14 were detected in LS conditions that affected days to heading. For GFD, twelve marker in NS and eleven markers in LS conditions were detected. The trait PH was found to be associated with eleven genomic regions in NS and twelve in LS conditions; SPP with seven regions in NS and six in LS conditions. Three markers were found associated with GNS in NS and five in LS conditions. Fourteen MTAs were found in NS and seven in LS conditions that affected TKW. Sixteen MTAs in NS and 17 MTAs in LS conditions were detected that control GY (Table 4). Manhattan plots for the studied traits are available as additional files 3-9: Figures S3 to S9. A map based on physical base pair distance of 139 markers (associated with studied traits) was constructed and an 11 Mb (752 to 763Mb) genomic region on chromosome 2A was associated with TKW; a very narrow distance of just 0.3 Mb (from 748.5 Mb to 748.2 Mb) was found associated with PH and GY under late sown conditions. A region spanning 10 Mb (465.5 to 475.2 Mb) on chromosome 1B is associated with GY, SPP and from 592.9 to 608.2 Mb (16 Mb) is linked with GY and GFD under LS conditions. A 27 Mb region on 4A was associated with PH and TKW in normal sown conditions. On chromosome 5D, a 2Mb (42 to 44Mb) region was detected that was associated with DH. A narrowed distance of 0.48 Mb (611.56 to 611.08 Mb) on chromosome 6A was linked with PH and SPP. Another noticeable 4Mb region which extends from 47 to 51Mb on 6B is responsible for controlling TKW in NS conditions. A genomic region (321.6 Mb to 735 Mb) on chromosome 7A was found to be of prime importance due to association with multiple agronomic traits including DH, TKW and GY traits (Fig 6). Effect of favorable alleles was estimated for all seven traits studied under LS conditions. The highest allelic effect (61%) was observed for days to heading under LS conditions (Fig 7). Genotypes (serial number 34, 109, 115, 120; additional file 1: Table S1) with nine favorable alleles exhibited the highest (> 0.06 kg/plot) grain yield. Favorable alleles for grain yield explained 45% variability. Increase in grain yield was observed as the number of favored allele increased additively (R^2^ = 0.45). Number of favored alleles in each genotype for seven traits under LS conditions is given in additional file 1: Table S1.

Seven MATs were selected on the basis of >0.3 MAF and their sequences were searched using Basic Local Alignment Search Tool (BLAST) through the link (https://plants.ensembl.org /Multi/Tools/Blast?db=core). With these seven sequences, 144 hits were found. Fourteen gene hits with greater than 90% sequence similarity were selected with their respective annotations (Table 5). Our BLAST results can be accessed with the link: <https://plants.ensembl.org/Multi/Tools/Blast/Ticket?tl=MSwRDAw72lU8IpJV>

**Discussion**

Source to sink ratio is disturbed when heat stress affects the photosynthetic apparatus due to alteration in carbon assimilatory processes [28] which causes reduction in yield and yield related agronomic traits. Hence the present study was designed to evaluate the heat resistance potential of 125 spring wheat genotypes under three different locations. In this detailed phenotypic experiment, the traits were examined under two sowing conditions (NS and LS) that provided the copious opportunity to observe the difference exhibited by a trait under normal and heat-stressed (late sown) conditions. Traits studied in different locations were averaged to understand the stability across the environment as performed by Ogbonnaya et al. [20]. In this way their combined contribution under overall environmental variation in the detection of associated genomic regions has been highlighted under normal and heat-stressed conditions. Thus our study showed the MTAs for normal and late sown conditions separately, so that actively participating genomic regions controlling the traits under heat stress can be emphasized.

After reviewing the previous literature, only those traits were selected for evaluation and eventually genome-wide mapped which were reportedly affected by the heat stress [16-17]. Significant reduction in all studied traits was observed due to heat stress. Except DH and PH, all traits were affected by genotype x environment interaction and their ranges show that the traits are quantitatively inherited, as has been in prior investigation [17] also showed such trends. Heritability estimates for the traits DH, SPP and GNS were lower in late sown than normal sown trials same as reported by Tiwari et al. [17] while GFD, PH, TKW and GY have higher heritability in late sown than normal sown conditions as shown by Paliwal et al. [16], similar heritability estimates for TKW have been reported by Pinto et al. [29].

Two-way analysis of variance showed acceptable ranges for CV% indicated that the design of the experiment is appropriate, similar trends in CV % have been shown by Ogbonnaya et al. [20]. Under heat-stressed conditions, GY is significantly affected by PH, GFD, SPP and GNS in a positive manner while PH significantly affected DH, SPP and TKW. Under heat stressed conditions DH is inversely related with SPP, GFD and GY as well while GFD if reduced that can cause ultimate reduction in the grain yield. Similar trends have also been reported by Paliwal et al. [16]. Early maturing wheat lines have maximum GFD that can cause an increase in GY.

The biplot was constructed to see the expression of genotypes in normal and heat-stressed conditions with respect to the studied traits. The biplot distinctly which genotypes performed well under normal conditions, under heat-stressed and under both. It has also been observed that the first principal component in LS conditions explains higher variability (30.14%) as than NS conditions (24.52%), and the same trend was followed by second principal component in NS and LS conditions. Variation among the genotypes has been well explained under heat-stressed conditions as compared to under normal conditions. Genotypes with good height were high yielding and those genotypes having high number of spikes per plant (SPP) have been found with high GNS under heat-stressed conditions and delayed heading caused shrinkage in GFD.

As proven by the LSD value for each trait, the effect of heat stress was significant, so the MTAs detected in both normal and heat-stressed conditions were underlined. The main focus of the study was to identify the genomic regions under normal and heat-stressed conditions so the heat susceptibility indices for the traits as calculated and mapped by some of the previous studies [16-17] was not the objective of this study. Mapping for heat susceptibility indices of the traits cannot detect those genomic regions specifically expressed under heat stress, so the main interest was to identify the MTAs under heat stress and to pinpoint the involvement of different chromosomal regions due to normal and heat-stressed conditions.

In the present trait DH was associated with six markers in normal and fourteen markers on 8 different chromosomes in heat-stressed conditions. Two markers on 1D and two markers on 5D along with three markers on 6B and three markers on 7A were identified as controlling the DH in LS conditions. Four markers, one on each of 2A, 4A, 5B and 6A were also identified meanwhile. Fifteen percent variation in DH has been explained under heat stressed conditions by the marker (S6A_71077435) on 6A; Ogbonnaya et al. [20] also reported a region on 6A with 7.48 R^2^ for DH. Using functional markers *VRN-A1*, *VRN-B1* and *VRN-D1* on chromosomes 5A, 5B and 5D Ogbonnaya et al. [20] explained the variation in DH. In the present study, four markers (one on 5B, three on 5D) explained 15% variation in DH.

Grain filling duration was controlled by the regions on some multiple chromosomes (Table 4) in NS and LS conditions. Above 12 % variation has been explained by most of the regions in both sowing conditions. Paliwal et al. [16] reported the distance between two markers (*Xgwm935-Xgwm1273*) on chromosome 2B long arm linked with GFD, later on Tiwari et al. [17] identified other QTLs for GFD were identified and most recently Ogbonnaya et al. [20] detected a region on 1B explaining 4.5% of GFD. Present study identified some GFD operating regions on 2B, 5B, 5D and 7D which validates previous [16-17] findings and also underlined some additional regions on chromosomes 3B and 4A. A region on chromosome 1B that explained 15% variation in GFD under heat-stressed conditions also corroborate with the findings of Ogbonnaya et al. [20].

Using DArTseq, plant height was mapped by Bellucci et al. [30] on chromosome 2A and 6A explaining 6 to 7% variation. Ogbonnaya et al. [20] reported a reported an MTA for PH on 5A with 4.85 R^2^. We report a 6Mb region on 5A (426 to 732Mb) associates with PH and explains 12 to 14% variation along with another locus on the same (5A) chromosome with the 23% R^2^. Under heat stress, there is another region of 1Mb (32Mb to 33Mb) on chromosome 3B that is associated with PH. In European winter wheat, using SSR, MTA on 6A for PH was reported [31-32]. In our study a genomic region on chromosome 6A at 212Mb is linked with PH in NS conditions but a region on the same (6A) chromosome ~400Mb away (at 611Mb) has also been detected controlling plant height under heat stress explaining 16% and 10% variation respectively. Spikes per plant was associated with genetic regions on 5A, 5B, 6A, 7A, 7B in NS conditions but in LS conditions the trait variation has been explained up to 14 % with regions on 1B, 4B and 7A. A two Mb spanned genetic region on 2D (72-74Mb) explained 12% variation in SPP under heat stress. Number of spikes per plant is a trait of prime importance for yield [33], so the information given by these MTAs can be used to predict the number of spikes under heat stress. For grain number per spike, in addition to the previously reported [20] region on 7B, we report MTAs for GNS on 3A and 5D under NS and on 2A, 2D, 6A and 6D under LS conditions with explained R^2^ from 10 to 19%.

Thousand kernel weight is reportedly governed by the regions on 5A, 6A [18], 1A, 1B, 2D, 5A, 6A [20]. Paliwal et al. [16] detected MTAs for TKW on 2B, 7B and 7D. Tiwari et al. (2013) also showed the MTAs for TKW in normal and heat-stressed conditions on 2B, 6B and 2B, 6A, 7D respectively. In the present research a 4Mb region on chromosome 6B uncovered by four GBS markers explained 12 to 14% variation in TKW, not only these results validating the previous findings as well as revealing the function of additional genomic region on 6B in NS conditions. But in heat-stressed conditions, a chromosomal location on 3B revealed by three GBS markers explained 10 to 13% variation in TKW. An MTA for TKW on 2B identified in this study is in agreement with the findings of Paliwal et al. [16] and Tiwari et al. [17]. Another set of three markers on 7A in the region from 718 to 735Mb explained 10 to 11% variation in TKW.

Grain yield is a complex quantitative trait and has MTAs spread over different chromosomes; in both NS and LS conditions, two MTAs were detected on chromosome 1A that explained 13% variation. Chromosome 1B has been reported [20] responsible for 7.55% explained variation while in our study we identified three markers on 1B which explained 13 to 16 % variation in GY under heat-stress conditions. In NS condition, two markers on 2B explained 12 to 13 % variation; Tiwari et al. [17] and Paliwal et al. [16] also reported QTLs on 2B for GY. Moreover, some MTAs on 3B, 5B, 6A, 6D, 7A, 7B and 7D have also been detected in this study for GY.

As argued by Paliwal et al. [16] in the heat stress studies, a chromosomal region on 2B is of prime importance. In the present study, 11 MTAs for different traits have been detected on 2B. Using reference genome, GBS-markers associated with the traits of interest were ordered on the basis of physical mega base pair distance and have been reported in this study in the form of a physical map as guided by Poland et al. [22].

**Conclusion**

To assess the thermo tolerance of the wheat germplasm, there is a dire need to highlight those regions involved in the adaptation to high temperature. This can be done by finding the association of genomic regions with the help of next generation SNP genotyping (such as GBS) so that the detected sequences can be utilized for the development of high throughput user friendly markers which may be used in marker assisted or genomic selection. Our study contributed in the effort of finding the genomic regions expressed during high temperature. Seven chromosomal regions with minor allele frequencies greater than 0.3 have been identified and their sequence was searched with BLAST which showed 14 gene hits with >90% similarity. The genotypes with high number of favorable alleles detected in this study can be utilized in wheat breeding programs.

**Material and Methods**

**Material**

Germplasm was comprised of 125 spring wheat genotypes (Additional file 1: Table S1) obtained from International Maize and Wheat Improvement Centre (CIMMYT). Field trials were conducted at three different locations in Pakistan; i) National Agriculture Research Center (NARC), Islamabad; ii) University of Sargodha, Sargodha and iii) Regional Agriculture Research Institute (RARI) Bahawalpur. Geographical coordinates for the experimental location at Islamabad (ISD) were 33o40ꞌ34ꞌꞌN 73o7ꞌ54ꞌꞌE, Sargodha (SGD) 32o7ꞌ50ꞌꞌN 72o40ꞌ50ꞌꞌE and Bahawalpur (BWP) 29o23ꞌ11ꞌꞌN 71o39ꞌ12ꞌꞌE. At each location, one complete set of germplasm was sown at the optimum time (during third week of November) considered as normal and the second complete set was sown late (during third week of December) to uncover the genotype response to high temperature during anthesis. The meteorological data has been presented in the additional file Figure S10.

**Phenotyping**

Alpha lattice design with two replications was followed for all the experiments. Plots of two meter two rows with 30cm row spacing were specified for each entry. Each trait under study was recorded separately for normal and late sown trials. Days to heading (DH) which is the period from sowing to the appearance of heads was noted when more than 50% of the plants of each genotype displayed heads at Zadoks stage 59 [34]. Grain filling duration (GFD) was recorded as the phase between heading and physiological maturity; the stage from sowing date to the time when green color of more than 50% of the spikes disappeared at Zadoks stage 89 [34], which is referred to as physiological maturity. After reaching physiological maturity, plant height (PH) in centimeters was measured with meter rule from the base of the plant up to the top of the spike excluding the awn length. Number of spikes per plants (SPP) was also estimated by taking the average of the number of spikes of five randomly sampled plants of each entry. Grain number per spike (GNS) was calculated, as it was the average of kernels in the main stem spikes of each of ten randomly selected plants from each entry. Grains of all spikes of ten randomly selected plants of each entry were bulked separately. Thousand kernel weight (TKW) was measured in grams by counting thousand grains randomly from each bulk using electrical weighing balance. Grain yield in kilograms for each entry was recorded after threshing whole two meter two rows plot.

**GBS-SNP genotyping**

All 125 lines were genotyped using genotyping-by-sequencing (GBS). DNA extraction kit from Prima Scientific (Bangkok, Thailand) was used to extract genomic DNA. GBS was performed following the Mascher et al. [35] protocol using two-enzyme (*MspI-PstI*) approach. GBS-SNPs were identified from sequence tags by aligning the sequence reads to the reference genome of Chinese Spring (CS) wheat. A pipeline of TASSEL 5 software was used for SNP calling against the whole wheat (CS) genome assembly (WGA 0.4, International Wheat Genome Sequencing Consortium). SNPs were called by setting minimum/maximum minor allele frequencies (MAF, 0.02 and 0.5) and minimum locus coverage was set at 0.2. Identified SNPs were named as “chromosome number_ physical position” *i.e.* 6D_13778811. The SNP markers with >10% missing data and > 20% heterozygosity were not considered for further analysis. The genotypic panel of 55954 SNP marker used in the study has average minor allele frequency 0.197; proportion of overall heterozygosity was 0.107 while the average missing proportion was 0.052.

**Statistical analysis**

By using the software Meta-R, best linear unbiased estimates (BLUEs) for three locations were calculated from restricted maximum likelihood (REML) analysis. The REML analysis was performed on results achieved in the alpha lattice experiments. Variance components were estimated with the software Statistix 8.1. Heritability (h^2^) was calculated for normal and late sown trials using the formula, h^2^= σ^2^g / σ^2^p ; where σ^2^g (σ^2^g=[(σ^2^genotypes- σ^2^error)/replicates]) is genotypic mean square and σ^2^p (σ^2^p = σ^2^g + σ^2^error) is phenotypic mean square. Biplot construction and correlation analysis for the traits in normal and late sown trials was performed using XLSTATS 2010 and the package “Corrplot” in R-software respectively [36].

To understand the genetic stratification of 125 genotypes, principal component analysis (PCA) was performed using their marker data and distance square matrix was obtained using built-in relatedness tab through TASSEL. This distance square matrix was used to explore the clusters in the panel with Ward’s method of agglomerative hierarchical clustering (AHC).

Linkage disequilibrium (LD) analysis was perform to obtain LD estimates expressed as r2 on sliding window of 100 markers by treating heterozygous calls as missing. To observe the LD decay, intra-chromosomal r2 was plotted against base pair distance using R-package ggplot2. Locally weighted scatter-plot smoother (LOWESS) function was used to draw a trend line for the detection of a threshold r2 below which relationship of any two pairs is to be considered not due to physical linkage. Marker pairs in significant (p-value <0.05) LD were further observed with respect to inter and intra-chromosomal aspect and pairs with <0.1 r^2^ were identified.

Polymorphism information content (PIC) and minor allele frequency (MAF) were calculated using the software Trait Analysis by Association, Evolution and Linkage (TASSEL 5.0) [37]. For the examined traits, calculated BLUEs were used for marker-trait association (MTAs). Genome-wide association study (GWAS) was performed using an optimally compressed mixed linear model (MLM) along with variance component estimation with P3D (population parameters previously determined) as implemented in TASSEL by Zhang et al. [38]. A kinship matrix (K) and five principal components (PC) as covariate were used [39] to run MLM (K + PCA) in TASSEL. The equation fitted in TASSEL 5.0 was: y = Xβ+Zµ+e , where y is the vector of observation for phenotypic values; β is an unknown vector with fixed effects including genetic marker and PC and µ is the unknown vector of random additive genetic effects for lines. To avoid the false positives, kinship matrix (K) was used in the model as an additive genetic effect for lines; X and Z are known matrices; and e is the vector which is unobserved of random residual. In this model µ and e are the vectors which are assumed to be normally distributed. False discovery rate (FDR) method was used to estimate the significance between marker and trait association [40] keeping a q-value cutoff of 0.05.

**List of abbreviations**

**AHC**: Agglomerative hierarchal clustering; **BLUEs**: Best linear unbiased estimations; **BWP**: Bahawalpur; **DArT**: Diversity array technology; **DH**: Days to heading; **FDR**: Falls discovery rate; **GBS**: Genotyping-by- sequencing; **GFD**: Grain filled duration; **GNS**: Grain number per spike; **GWAS**: Genome-wide association study; **GY**: Grain yield; **ISD**: Islamabad; **LD**: Linkage disequilibrium; **LS**: Late sown; **MAF**: Minor/minimum allele frequency; **Mb**: Mega base; **MTA**: Marker-trait association; **NARC**: National agriculture research center; **NS**: Normal sown; **PCA**: Principal component analysis; **PH**: Plant height; **PIC**: Polymorphism information contents; **QTL**: Quantitative trait locus; **RARI**: Regional agriculture research institute; **REML**: Restricted maximum likelihood; **SGD**: Sargodha; **SNP**: Single nucleotide polymorphism; **SPP**: Spikes per plant; **TKW**: Thousand kernel weight; **WGA**: Whole genome assembly

**Declarations**

**Ethics approval and consent to participate**

We declare that these experiments within the ethical standards and legislations in Pakistan.

**Consent for publication**

National and international guidelines were followed during the collection of plant material.

**Availability of data and material**

The phenotypic data of the current study is available in the Additional file 1: Table S1. Any other datasets used and/or analyzed are available upon request through email to the corresponding author.

**Competing interest**

The authors declare that they have no competing interest

**Funding**

No funding was released for this research work**.**

**Authors' contributions**

MJ performed the experiment and wrote the paper. AA performed the multivariate data analysis. AG^2^ performed genotyping-by-sequencing. AG^3^ performed the field planting and sample collection of wheat lines. AAN collected the field data. AMHI formulated the univariate statistical analysis. NHN and NAY contributed in revisions. AMK planned the experiment. All the authors have read and approved the final manuscript.

**Acknowledgements**

We thank P. St Amand, A. Bernardo and G. Bai (United States Department of Agriculture-Agricultural Research Service, Hard Winter Wheat Genetics Research Unit, Manhattan, KS 66506, USA) for supervising the genotyping-by-sequencing and Manzoor Hussain (Regional Agricultural Research Institute (RARI) Bahawalpur, Pakistan) for conducting the field trials at Bahawalpur.

**References**

1. Borlaug NE, Dowswell CR. Feeding a world of ten billion people: a 21st century challenge. In R Tuberosa, RL Phillips, M Gale, Eds, Proceedings of the International Congress in theWake of the Double Helix: From the Green Revolution to the Gene Revolution, 27–31 May 2003, Bologna, Italy. Avenue Media, Bologna, Italy; 2005. p 3–23.

2. Stratonovitch P, Semenov MA. Heat tolerance around flowering in wheat identified as a key trait for increased yield potential in Europe under climate change. J Exp Bot. 2015; 66(12):3599–3609. https://doi.org/10.1093/jxb/erv070.

3. IPCC. Summary for Policymakers of Climate Change 2007: The Physical Science Basis Contribution of Woring Group I to the Fourth Assessment Report of the Inter-governmental Panel on Climate Change. UK, Cambridge University Press, Cambridge.

4. Ray DK, Mueller ND, West PC, Foley JA. Yield trends are insufficient to double global crop production by 2050. 2013; PLoS ONE 8:e66428.

5. Joshi AK, Mishra B, Chatrath R, Ferrara GO, Singh RP. Wheat improvement in India: present status, emerging challenges and future prospects. Euphytica. 2007; 157:431-446.

6. Reynolds MP, Nagarajan S, Razzaque MA, Ageeb OAA. Breeding for adaptation to environmental factors, heat tolerance. In: Reynolds, M.P., Ortiz- Monasterio, I., McNab, A. (Eds.), Application of Physiology in Wheat Breeding. CIMMYT, Mexico, 2001. p.124-125.

7. Asseng S, Ewert F, Martre P, Rötter RP, Lobell DB. Rising temperatures reduce global wheat production. Nat Clim Change. 2015; 5:143–147.

8. Wardlaw IF, Dawson IA, Munibi P. The tolerance of wheat to high tem- perature during grain reproductive growth. I. Survey procedures and general response patterns. Aust J Agric Res. 1989; 40:1–13.

9. Prasad PVV, Pisipati SR, Ristic Z, Bukovnik U, Fritz AK. Impact of nighttime temperature on physiology and growth of spring wheat. Crop Sci. 2008; 48(6):2372-2380.

10. Hays DB, Do JH, Mason RE, Morgan G, Finlayson SA. Heat stress induced ethylene production in developing wheat grains induces kernel abortion and increased maturation in a susceptible cultivar. Plant Sci. 2007; 172:1113-1123.

11. Rane J, Pannu RK, Sohu VS, Saini RS, Mishra B, Shoran J, et al. Performance of yield and stability of advanced wheat genotypes under heat stressed environments of Indo-Gangetic Plains. Crop Sci. 2007; 47: 1561–1573.

12. Stone PJ, Nicolas ME. Wheat cultivars vary widely in their responses of grain yield and quality to short periods of post-anthesis heat stress. Aust J Plant Physiol. 1994; 21:887-900.

13. Mondal S, Rutkoski JE, Velu G, Singh PK, Crespo-Herrera LA, et al. Harnessing diversity in wheat to enhance grain yield, climate resilience, disease and insect pest resistance and nutrition through conventional and modern breeding approaches. Front Plant Sci. 2016; 7: 991. https://doi.org/10.3389/FPLS.2016.00991.

14. Yang J, Sears RG, Gill BS, Paulsen GM. Quantitative and molecular characterization of heat tolerance in hexaploid wheat. Euphytica. 2002; 126:275-282.

15. Mohammadi V, Zali AA, Bihamta MR. Mapping QTLs for Heat Tolerance in Wheat. J Agr Sci Tech. 2008; 10:261-267.

16. Paliwal R, Röder MS, Kumar U, Srivastava JP, Joshi AK. QTL mapping of terminal heat tolerance in hexaploid wheat (*T. aestivum* L.). Theor Appl Genet. 2012; 125(3):561-575. <https://doi.org/10.1007/s00122-012-1853-3>.

17. Tiwari C, Wallwork H, Kumar U, Dhari R, Arun B, Mishra VK, et al. Molecular mapping of high temperature tolerance in bread wheat adapted to the Eastern Gangetic Plain region of India. Field Crops Res. 2013; 154: 201-210. https:// doi.org/ 10.1016/j. fcr. 2013.08.004.

18. Sukumaran S, Dreisigacker S, Lopes M, Chavez P, Reynolds M.P. Genome-wide association study for grain yield and related traits in an elite spring wheat population grown in temperate irrigated environments. Theor Appl Genet. 2015; 128(2):353-363. https:// doi. org/10.1007/s00122-014-2435-3

19. Rasheed A, Hao Y, Xia X, Khan A, Xu Y, Varshney RK, et al. Crop Breeding Chips and Genotyping Platforms: Progress, Challenges, and Perspectives. Mol Plant. 2017; 10(8), 1047–1064. https://doi.org/10.1016/j.molp.2017.06.008.

20. Ogbonnaya FC, Rasheed A, Okechukwu EC, Jighly A, Makdis F, Wuletaw T, et al. Genome-wide association study for agronomic and physiological traits in spring wheat evaluated in a range of heat prone environments. Theor Appl Genet. 2017; 130(9):1819-1835. https://doi.org/10.1007/s00122-017-2927-z.

21. Elshire RJ, Glaubitz JC, Sun Q, Poland JA, Kawamoto K, Buckler ES, et al. A robust, simple genotyping-by-sequencing (GBS) approach for high diversity species. PloS ONE. 2011; 6(5): e19379. https:// doi.org/ 10.1371/ journal. pone.0019379.

22. Poland J.A, Rife TW. Genotyping-by-Sequencing for Plant Breeding and Genetics. The Plant Genome Journal, 2012; 5(3):92. <https://doi.org/10.3835/> plantgenome2012. 05.0005.

23. Sukumaran S, Lopes M, Dreisigacker S, Reynolds M. Genome wide association mapping for grain weight in spring wheat across multiple environments. Conference Paper, (March), 2017; 20-27.

24. Mwadzingeni L, Shimelis H, Rees DJG, Tsilo TJ. Genome-wide association analysis of agronomic traits in wheat under drought-stressed and non-stressed conditions. PLoS ONE, 2017; 12(2):1-13. https://doi.org/10.1371/journal.pone.0171692.

25. Guo Z, Chen D, Alqudah AM, Röder MS, Ganal MW, Schnurbusch T. Genome‐wide association analyses of 54 traits identified multiple loci for the determination of floret fertility in wheat. New Phytol. 2017; 214(1):257-270. <https://doi>.org/ 10.1111/nph.14342.

26. Arora S, Singh N, Kaur S, Bains NS, Uauy C, Poland J, et al. Genome-Wide Association Study of Grain Architecture in Wild Wheat *Aegilops tauschii*. Front Plant Sci. 2017; 8:886. https://doi.org/10.3389/fpls.2017.00886.

27. Li J, Rasheed A, Guo Q, Dong Y, Liu J, Xia X, et al. Genome-wide association mapping of starch granule size distribution in common wheat. J Cereal Sci. 2017; 77: 211-218. <https://doi.org/10.1016/j.jcs.2017.08.016>.

28. Kumar S, Kumari P, Kumar U, Grover M, Singh AK, Singh R, Sengar RS. (). Molecular approaches for designing heat tolerant wheat. J Plant Biochem Biot. 2013; 22(4):359-371.

29. Pinto RS, Reynolds MP, Mathews KL, McIntyre CL, Olivares-Villegas J, Chapman SC. Heat and drought adaptive QTL in a wheat population designed to minimize confounding agronomic effects. Theor Appl Genet. 2010; 121:1001–1021.

30. Bellucci A, Torp AM, Bruun S, Magid J, Andersen SB, Rasmussen SK. Association Mapping in Scandinavian Winter Wheat for Yield, Plant Height, and Traits Important for Second-Generation Bioethanol Production. Front Plant Sci. 6(November), 2015; 1–12. https://doi.org/10.3389/fpls.2015.01046.

31. Zanke CD, Ling J, Plieske J, Kollers S, Ebmeyer E, Korzun V, et al. Analysis of main effect QTL for thousand grain weight in European winter wheat (*Triticum aestivum* L.) by genome-wide association mapping. Front Plant Sci. 6(September): 2015; 1–14.

32. Wurschum T, Langer SM, Longin CFH. Genetic control of plant height in European winter wheat cultivars. Theor Appl Genet. 2015; 128(5):865–874.doi:10.1007/s00122-015-2476-2

33. Chen GF, Wu RG, Li DM, Yu HX, Deng Z, Tian JC. Genome wide association study for seeding emergence and tiller number using SNP markers in an elite winter wheat population. J Genet. 2017; 96(1):177-186. https://doi.org/10.1007/s12041-016-0731-1.

34. Zadoks JC, Chang TT, Konzak CF. A decimal code for growth stages of cereals. Weed Res. 1974; 14:415–421.

35. Mascher M, Wu S, Amand PS, Stein N, Poland J. Application of Genotyping-by-Sequencing on Semiconductor Sequencing Platforms: A Comparison of Genetic and Reference-Based Marker Ordering in Barley. PLoS ONE 2013; 8(10):e76925. doi:10.1371/journal.pone.0076925.

36. Wei T, Simko V, Levy M, Xie Y, Jin Y, Zemla J. (2017). Package ‘corrplot’. Statistician 2017;  56:316-324.

37. Bradbury PJ, Zhang Z, Kroon DE, Casstevens TM, Ramdoss Y, Buckler ES. TASSEL: software for association mapping of complex traits in diverse samples. Bioinformatics 2007; 23:2633-2635.

38. Zhang Z, Ersoz E, Lai CQ, Todhunter RJ, Tiwari HK, Gore MA, et al. Mixed linear model approach adapted for genome-wide association studies. Nat Genet. 2010; 42(4):355-360. https://doi.org/10.1038/ng.546.

39. Yu J, Pressoir G, Briggs WH, Vroh I, Bi M, Yamasaki JF, et al. A unified mixed-model method for association mapping that accounts for multiple levels of relatedness. Nat Genet. 2006; 38: 203-208.

40. Benjamini Y, Hochberg Y. Controlling the false discovery rate: a practical and powerful approach to multiple testing. J R Stat Soc B. 1995; 57:289-300.

**Table 1.** Description of studied traits at three locations (Islamabad: ISD, Sargodha: SGD and Bahawalpur: BWP) along with over all mean, mean squares, percent reduction due to late sown and heritability

| **Trait** | **Location** | **Treatment** | **Mean** | **SE** | **Min.** | **Max.** | **Overall** | **Mean** | **MS** | **h^2^** |
| --- | --- | --- | --- | --- | --- | --- | --- | --- | --- | --- |
| **DH** | ISD | Normal | 126 | 0.39 | 110 | 133 | Normal | 119 | 19.7*** | 0.86 |
|  |  | Late | 88 | 0.18 | 84 | 93 | Late | 82 | 8.65^ns^ | 0.71 |
|  | SGD | Normal | 123 | 0.24 | 118 | 130 | LSD | 0.5 |  |  |
|  |  | Late | 81 | 0.09 | 78 | 82 | Reduction% | ISD | SGD | BWP |
|  | BWP | Normal | 108 | 0.31 | 97 | 113 | Mean | 30 | 34 | 28 |
|  |  | Late | 78 | 0.44 | 64 | 86 | Min-Max | 24-30 | 34-37 | 24-34 |
| **GFD** | ISD | Normal | 34 | 0.31 | 26 | 42 | Normal | 35 | 9.82^ns^ | 0.74 |
|  |  | Late | 27 | 0.35 | 18 | 38 | Late | 28 | 13.39^ns^ | 0.76 |
|  | SGD | Normal | 37 | 0.2 | 31 | 42 | LSD | 0.43 |  |  |
|  |  | Late | 33 | 0.17 | 29 | 38 | Reduction% | ISD | SGD | BWP |
|  | BWP | Normal | 35 | 0.32 | 24 | 45 | Mean | 22 | 9 | 34 |
|  |  | Late | 23 | 0.4 | 14 | 35 | Min-Max | 9-31 | 06-10 | 22-42 |
| **PH (cm)** | ISD | Normal | 93.2 | 0.56 | 75.7 | 109.3 | Normal | 87.9 | 47.83*** | 0.83 |
|  |  | Late | 69.53 | 0.61 | 51 | 90.7 | Late | 65.7 | 93.68*** | 0.88 |
|  | SGD | Normal | 85.77 | 0.55 | 72 | 101.5 | LSD | 0.78 |  |  |
|  |  | Late | 59.74 | 0.69 | 40 | 80 | Reduction% | ISD | SGD | BWP |
|  | BWP | Normal | 84.75 | 0.46 | 72 | 96 | Mean | 25 | 30 | 20 |
|  |  | Late | 67.83 | 0.69 | 48.67 | 88.67 | Min-Max | 17-33 | 21-44 | 8-32 |
| **SPP** | ISD | Normal | 8 | 0.14 | 5 | 16 | Normal | 9 | 2.79*** | 0.82 |
|  |  | Late | 5 | 0.08 | 3 | 8 | Late | 5 | 2.03^ns^ | 0.78 |
|  | SGD | Normal | 10 | 0.15 | 7 | 14 | LSD | 0.17 |  |  |
|  |  | Late | 5 | 0.11 | 2 | 8 | Reduction% | ISD | SGD | BWP |
|  | BWP | Normal | 8 | 0.08 | 6 | 11 | Mean | 41 | 51 | 52 |
|  |  | Late | 4 | 0.15 | 1 | 8 | Min-Max | 40-50 | 43-71 | 27-83 |
| **GNS** | ISD | Normal | 53 | 0.81 | 24 | 74 | Normal | 59 | 152.8*** | 0.86 |
|  |  | Late | 41 | 0.92 | 10 | 60 | Late | 41 | 100.8** | 0.81 |
|  | SGD | Normal | 59 | 1.01 | 30 | 85 | LSD | 1.04 |  |  |
|  |  | Late | 41 | 0.7 | 24 | 60 | Reduction% | ISD | SGD | BWP |
|  | BWP | Normal | 64 | 0.84 | 40 | 87 | Mean | 23 | 31 | 34 |
|  |  | Late | 42 | 0.77 | 22 | 62 | Min-Max | 19-58 | 20-29 | 29-45 |
| **TKW (g)** | ISD | Normal | 42.26 | 0.52 | 26.07 | 54 | Normal | 40.6 | 46.78** | 0.81 |
|  |  | Late | 33.2 | 0.62 | 14.87 | 48.31 | Late | 28.8 | 43.68*** | 0.85 |
|  | SGD | Normal | 41.99 | 0.5 | 19 | 51.87 | LSD | 0.64 |  |  |
|  |  | Late | 27.77 | 0.41 | 17 | 44.36 | Reduction% | ISD | SGD | BWP |
|  | BWP | Normal | 37.65 | 0.6 | 22.32 | 54.55 | Mean | 21 | 34 | 32 |
|  |  | Late | 25.46 | 0.41 | 14.79 | 41.2 | Min-Max | 11-43 | 11-14 | 24-34 |
| **GY (Kg/plot)** | ISD | Normal | 0.16 | 0.01 | 0.01 | 0.34 | Normal | 0.14 | 0.0037^ns^ | 0.72 |
|  |  | Late | 0.05 | 0.002 | 0.01 | 0.17 | Late | 0.03 | 100.8** | 0.81 |
|  | SGD | Normal | 0.15 | 0.01 | 0.02 | 0.39 | LSD | 0.006 |  |  |
|  |  | Late | 0.04 | 0.002 | 0.001 | 0.13 | Reduction% | ISD | SGD | BWP |
|  | BWP | Normal | 0.11 | 0.01 | 0.02 | 0.56 | Mean | 66 | 76 | 74 |
|  |  | Late | 0.03 | 0.002 | 0.002 | 0.13 | Min-Max | 17-49 | 68-95.6 | 77-98 |

Days to heading: DH, grain filled duration: GFD, plant height: PH, spikes per plant: SPP, grain number per spike: GNS, thousand kernel weight: TKW and grain yield per plot: GY

**Table 2.** Mean squares of studied traits from two-way analysis of variance

| SOV | df | DH | GFD | PH | SPP | GNS | TKW | GY |
| --- | --- | --- | --- | --- | --- | --- | --- | --- |
| Genotype (G) | 124 | 7.25E+05** | 15.55*** | 96.47*** | 2.84*** | 186.94*** | 74.03*** | 1.2*** |
| Location (L) | 2 | 5.78E+08*** | 2088.15*** | 4677.76*** | 198.97*** | 2158.05*** | 2389.6*** | 3.57*** |
| Treat (T) | 1 | 1.05E+10*** | 11097.63*** | 92463.63*** | 3555.58*** | 58132.81*** | 26224.08*** | 71.81*** |
| G X L | 248 | 3.08E+05ns | 13.82*** | 34.32ns | 2*** | 90.25*** | 34.37*** | 0.09*** |
| G X T | 124 | 6.21E+05* | 7.63ns | 45.034** | 1.98** | 66.67* | 16.43ns | 0.08*** |
| L X T | 2 | 1.74E+08*** | 1181.97*** | 1395.99*** | 53.16*** | 1353.50*** | 423.86*** | 1.09*** |
| Error | 248 | 4.62E+05 | 9.07 | 29.71 | 1.341 | 52.41 | 20.04 | 0.05 |
| CV% |  | 6.5 | 9.54 | 7.1 | 16.94 | 14.15 | 12.89 | 18.52 |

DH: days to heading, GFD: grain filled duration, PH: plant height, SPP: spikes per plant, GNS: grain number per spike, TKW: thousand kernel weight and GY: grain yield. *p-value <0.05, **p-value < 0.01, ***p-value <0.001

**Table 3.** Genome-wide details of 55954 markers along with polymorphism information contents and marker diversity

|  | Chromosome | Markers | Diversity | PIC |
| --- | --- | --- | --- | --- |
| **Genome A** | 1 | 2388 | 0.34 | 0.29 |
|  | 2 | 3185 | 0.34 | 0.29 |
|  | 3 | 2714 | 0.32 | 0.28 |
|  | 4 | 2684 | 0.33 | 0.28 |
|  | 5 | 2473 | 0.32 | 0.28 |
|  | 6 | 2352 | 0.32 | 0.27 |
|  | 7 | 3728 | 0.32 | 0.27 |
|  |  | **19524†** | **0.33‡** | **0.28‡** |
| **Genome B** | 1 | 2908 | 0.32 | 0.27 |
|  | 2 | 4248 | 0.33 | 0.28 |
|  | 3 | 3728 | 0.32 | 0.27 |
|  | 4 | 1906 | 0.32 | 0.28 |
|  | 5 | 3504 | 0.32 | 0.28 |
|  | 6 | 3354 | 0.32 | 0.27 |
|  | 7 | 3511 | 0.31 | 0.26 |
|  |  | **23159** | **0.32** | **0.27** |
| **Genome D** | 1 | 1686 | 0.33 | 0.29 |
|  | 2 | 2353 | 0.32 | 0.28 |
|  | 3 | 1992 | 0.31 | 0.27 |
|  | 4 | 1180 | 0.33 | 0.29 |
|  | 5 | 1811 | 0.33 | 0.29 |
|  | 6 | 1777 | 0.32 | 0.28 |
|  | 7 | 2472 | 0.32 | 0.28 |
|  |  | **13271** | **0.32** | **0.28** |
| Total |  | **55954** | **0.32§** | **0.28§** |

PIC 0.6 ≥ 0.5 = 1561 markers; 0.7 ≥ 0.6 PIC = 1260 markers and ≥ 0.7 PIC = 213 markers

**†** : Total Value ; **‡:** Mean value of sub-genome; **§:** Mean value across the whole genome A, B and D.

**Table 4. Marker-trait associations found for normal and late sown conditions keeping significant threshold p-value at 3.00 (-log10)**

| Trait |  | Marker | Chr | Pos (Mbs) | Allele | MAF | -log10(p) | Marker R^2^ |
| --- | --- | --- | --- | --- | --- | --- | --- | --- |
| Days to heading (DH) | NS | S2A_573947917 | 2A | 573.95 | A/G | 0.314 | 3.51 | 0.15 |
|  |  | S2B_15783383 | 2B | 15.78 | A/T | 0.253 | 3.38 | 0.12 |
|  |  | S3D_443442947 | 3D | 443.44 | G/T | 0.079 | 3.33 | 0.13 |
|  |  | S5D_42549600 | 5D | 42.55 | G/A | 0.365 | 3.56 | 0.15 |
|  |  | S7B_480230158 | 7B | 480.23 | T/C | 0.057 | 3.06 | 0.1 |
|  |  | S7D_6002850 | 7D | 6 | C/T | 0.48 | 3.34 | 0.15 |
|  | LS | S1D_416730006 | 1D | 416.73 | T/C | 0.162 | 3.16 | 0.14 |
|  |  | S1D_417084381 | 1D | 417.08 | C/G | 0.193 | 3.13 | 0.11 |
|  |  | S2A_43211220 | 2A | 43.21 | T/C | 0.252 | 3.22 | 0.14 |
|  |  | S4A_610095520 | 4A | 610.1 | G/A | 0.115 | 3.29 | 0.14 |
|  |  | S5B_586352552 | 5B | 586.35 | G/A | 0.497 | 3.36 | 0.15 |
|  |  | S5D_44161505 | 5D | 44.16 | G/A | 0.288 | 3.31 | 0.13 |
|  |  | S5D_552389328 | 5D | 552.39 | T/C | 0.185 | 3.29 | 0.13 |
|  |  | S6A_71077435 | 6A | 71.08 | T/C | 0.353 | 3.17 | 0.15 |
|  |  | S6B_296392848 | 6B | 296.39 | C/T | 0.121 | 3.27 | 0.13 |
|  |  | S6B_557614637 | 6B | 557.61 | C/A | 0.382 | 3.44 | 0.11 |
|  |  | S6B_700058360 | 6B | 700.06 | G/A | 0.113 | 3.58 | 0.15 |
|  |  | S7A_3066534 | 7A | 3.07 | T/G | 0.306 | 3.08 | 0.12 |
|  |  | S7A_321565067 | 7A | 321.57 | G/A | 0.066 | 3.05 | 0.12 |
|  |  | S7A_571203933 | 7A | 571.2 | T/A | 0.104 | 3.13 | 0.13 |
| Grain filling duration (GFD) | NS | S1A_48746498 | 1A | 48.75 | T/G | 0.215 | 3 | 0.13 |
|  |  | S2B_778750293 | 2B | 778.75 | G/T | 0.155 | 3.37 | 0.14 |
|  |  | S3B_627922 | 3B | 0.63 | A/G | 0.195 | 3.35 | 0.14 |
|  |  | S3B_792848261 | 3B | 792.85 | C/T | 0.103 | 3.18 | 0.14 |
|  |  | S3B_819163184 | 3B | 819.16 | A/C | 0.19 | 3.39 | 0.14 |
|  |  | S4D_441092051 | 4D | 441.09 | G/A | 0.082 | 3.14 | 0.13 |
|  |  | S5B_498781903 | 5B | 498.78 | G/A | 0.223 | 3.99 | 0.16 |
|  |  | S5D_82195139 | 5D | 82.2 | G/A | 0.064 | 3.06 | 0.12 |
|  |  | S5D_193133124 | 5D | 193.13 | C/G | 0.087 | 3.3 | 0.14 |
|  |  | S7A_17544272 | 7A | 17.54 | T/C | 0.43 | 3.09 | 0.13 |
|  |  | S7B_603999358 | 7B | 604 | C/T | 0.053 | 3.59 | 0.16 |
|  |  | S7D_28710742 | 7D | 28.71 | G/T | 0.052 | 3.03 | 0.1 |
|  | LS | S1B_608238167 | 1B | 608.24 | G/A | 0.141 | 3.18 | 0.15 |
|  |  | S1D_79799048 | 1D | 79.8 | A/T | 0.068 | 4.03 | 0.13 |
|  |  | S2B_10582115 | 2B | 10.58 | A/C | 0.127 | 3.07 | 0.13 |
|  |  | S2B_425762009 | 2B | 425.76 | G/T | 0.279 | 3.33 | 0.14 |
|  |  | S4A_610095520 | 4A | 610.1 | G/A | 0.115 | 3.24 | 0.14 |
|  |  | S4A_627995501 | 4A | 628 | G/A | 0.274 | 3.4 | 0.14 |
|  |  | S5A_551669444 | 5A | 551.67 | G/A | 0.17 | 3.13 | 0.13 |
|  |  | S6B_92246917 | 6B | 92.25 | A/T | 0.456 | 3.58 | 0.14 |
|  |  | S6B_256489407 | 6B | 256.49 | G/A | 0.08 | 3 | 0.13 |
| Plant height (PH) | NS | S3B_824599939 | 3B | 824.6 | C/T | 0.347 | 3.16 | 0.14 |
|  |  | S3D_12741374 | 3D | 12.74 | G/T | 0.083 | 3.9 | 0.17 |
|  |  | S4A_710817792 | 4A | 710.82 | C/T | 0.059 | 4.98 | 0.18 |
|  |  | S4B_95449920 | 4B | 95.45 | G/A | 0.165 | 3.01 | 0.12 |
|  |  | S5A_14254129 | 5A | 14.25 | G/A | 0.057 | 4.4 | 0.23 |
|  |  | S5A_426002537 | 5A | 426 | G/A | 0.23 | 3.48 | 0.14 |
|  |  | S5A_432036515 | 5A | 432.04 | G/A | 0.142 | 3.28 | 0.12 |
|  |  | S6A_212352003 | 6A | 212.35 | A/T | 0.072 | 3.65 | 0.16 |
|  |  | S7A_163318525 | 7A | 163.32 | G/T | 0.057 | 3.2 | 0.1 |
|  |  | S7A_618498297 | 7A | 618.5 | T/C | 0.148 | 3.09 | 0.09 |
|  |  | S7A_643002715 | 7A | 643 | G/A | 0.223 | 3.01 | 0.09 |
|  | LS | S1B_687868186 | 1B | 687.87 | A/T | 0.49 | 3.82 | 0.14 |
|  |  | S2A_14756019 | 2A | 14.76 | A/G | 0.055 | 3.52 | 0.13 |
|  |  | S2A_748204192 | 2A | 748.2 | A/C | 0.358 | 3.3 | 0.12 |
|  |  | S2B_9131487 | 2B | 9.13 | T/G | 0.054 | 3.03 | 0.11 |
|  |  | S2B_18494584 | 2B | 18.49 | A/T | 0.088 | 3.07 | 0.11 |
|  |  | S2B_770220840 | 2B | 770.22 | G/C | 0.446 | 4.21 | 0.16 |
|  |  | S3B_32471134 | 3B | 32.47 | A/G | 0.487 | 3.16 | 0.12 |
|  |  | S3B_33202313 | 3B | 33.2 | A/G | 0.29 | 3.51 | 0.13 |
|  |  | S3B_375363377 | 3B | 375.36 | G/A | 0.051 | 3.86 | 0.14 |
|  |  | S5A_625839432 | 5A | 625.84 | C/T | 0.055 | 3.58 | 0.14 |
|  |  | S6A_611557056 | 6A | 611.56 | C/T | 0.253 | 3.37 | 0.1 |
|  |  | S7A_6229645 | 7A | 6.23 | G/A | 0.089 | 3.68 | 0.14 |
| Spikes per plan (SPP) | NS | S5A_593094332 | 5A | 593.09 | G/A | 0.197 | 3.09 | 0.13 |
|  |  | S5B_62834860 | 5B | 62.83 | A/G | 0.347 | 3.03 | 0.12 |
|  |  | S5B_411753477 | 5B | 411.75 | G/C | 0.314 | 3.68 | 0.15 |
|  |  | S6A_611078199 | 6A | 611.08 | A/C | 0.131 | 3.04 | 0.09 |
|  |  | S7A_695820062 | 7A | 695.82 | A/G | 0.322 | 3.07 | 0.13 |
|  |  | S7B_687824893 | 7B | 687.82 | A/G | 0.352 | 3.16 | 0.13 |
|  |  | S7B_700803008 | 7B | 700.8 | A/G | 0.313 | 3.44 | 0.11 |
|  | LS | S1B_475270285 | 1B | 475.27 | G/T | 0.195 | 3.43 | 0.14 |
|  |  | S2D_72213516 | 2D | 72.21 | G/A | 0.131 | 3.17 | 0.12 |
|  |  | S2D_73405773 | 2D | 73.41 | C/T | 0.128 | 3.12 | 0.12 |
|  |  | S2D_74859293 | 2D | 74.86 | C/T | 0.135 | 3.65 | 0.12 |
|  |  | S4B_663622013 | 4B | 663.62 | C/T | 0.155 | 4.06 | 0.14 |
|  |  | S7A_69855692 | 7A | 69.86 | G/C | 0.12 | 3.28 | 0.12 |
| Grain numbers per spike (GNS) | NS | S3A_730295762 | 3A | 730.3 | T/G | 0.169 | 3.02 | 0.1 |
|  |  | S5D_503657305 | 5D | 503.66 | A/G | 0.431 | 3.27 | 0.14 |
|  |  | S7B_687521301 | 7B | 687.52 | T/C | 0.269 | 3.86 | 0.16 |
|  | LS | S2A_1050029 | 2A | 1.05 | A/G | 0.412 | 3.81 | 0.16 |
|  |  | S2A_575379774 | 2A | 575.38 | C/T | 0.142 | 3.18 | 0.12 |
|  |  | S2D_12870959 | 2D | 12.87 | C/G | 0.234 | 3.02 | 0.13 |
|  |  | S6A_615812205 | 6A | 615.81 | T/G | 0.132 | 3.2 | 0.12 |
|  |  | S6D_137788118 | 6D | 137.79 | G/A | 0.13 | 4.35 | 0.19 |
| Thousand kernel weight (TKW) | NS | S1B_526812249 | 1B | 526.81 | G/A | 0.228 | 3.2 | 0.13 |
|  |  | S2A_752870462 | 2A | 752.87 | A/G | 0.219 | 3.25 | 0.13 |
|  |  | S2B_235163611 | 2B | 235.16 | T/G | 0.099 | 3.03 | 0.12 |
|  |  | S4A_733664972 | 4A | 733.66 | C/T | 0.134 | 3.65 | 0.15 |
|  |  | S4A_737882127 | 4A | 737.88 | T/G | 0.331 | 3.15 | 0.12 |
|  |  | S6A_453869891 | 6A | 453.87 | A/G | 0.07 | 3.31 | 0.1 |
|  |  | S6B_47885327 | 6B | 47.89 | C/T | 0.281 | 3.42 | 0.14 |
|  |  | S6B_48222896 | 6B | 48.22 | C/T | 0.156 | 3.52 | 0.11 |
|  |  | S6B_48349083 | 6B | 48.35 | T/C | 0.141 | 3.07 | 0.12 |
|  |  | S6B_51093265 | 6B | 51.09 | A/C | 0.153 | 3.84 | 0.12 |
|  |  | S7A_646503003 | 7A | 646.5 | G/A | 0.059 | 3.08 | 0.09 |
|  |  | S7A_718192191 | 7A | 718.19 | T/C | 0.355 | 3.11 | 0.1 |
|  |  | S7A_732742920 | 7A | 732.74 | A/G | 0.143 | 3.44 | 0.11 |
|  |  | S7A_735390023 | 7A | 735.39 | A/G | 0.204 | 3.35 | 0.1 |
|  | LS | S2A_763944016 | 2A | 763.94 | G/A | 0.352 | 3.09 | 0.13 |
|  |  | S3B_220267045 | 3B | 220.27 | G/A | 0.202 | 3.43 | 0.11 |
|  |  | S3B_577609492 | 3B | 577.61 | A/G | 0.165 | 3.07 | 0.1 |
|  |  | S5B_648308504 | 5B | 648.31 | C/A | 0.22 | 3.13 | 0.13 |
|  |  | S6A_453869891 | 6A | 453.87 | A/G | 0.07 | 3.07 | 0.1 |
|  |  | S6B_680699350 | 6B | 680.7 | C/T | 0.223 | 4.33 | 0.18 |
|  |  | S7A_720839381 | 7A | 720.84 | T/C | 0.299 | 3.06 | 0.1 |
| Grain yield (GY) | NS | S1A_22415204 | 1A | 22.42 | G/T | 0.232 | 3.04 | 0.13 |
|  |  | S1B_465548215 | 1B | 465.55 | A/C | 0.169 | 3.1 | 0.13 |
|  |  | S1D_351086570 | 1D | 351.09 | C/T | 0.421 | 3.29 | 0.14 |
|  |  | S2B_43278561 | 2B | 43.28 | T/C | 0.076 | 3.54 | 0.12 |
|  |  | S2B_79870855 | 2B | 79.87 | G/A | 0.107 | 3.68 | 0.12 |
|  |  | S2B_753442043 | 2B | 753.44 | G/C | 0.439 | 3.18 | 0.13 |
|  |  | S3A_661975132 | 3A | 661.98 | C/A | 0.129 | 3.02 | 0.12 |
|  |  | S3B_285767866 | 3B | 285.77 | C/T | 0.247 | 3.09 | 0.14 |
|  |  | S3B_697598464 | 3B | 697.6 | C/A | 0.18 | 3.4 | 0.14 |
|  |  | S5B_310279281 | 5B | 310.28 | C/A | 0.084 | 3.03 | 0.12 |
|  |  | S6A_340738287 | 6A | 340.74 | T/C | 0.326 | 3.2 | 0.13 |
|  |  | S6D_3422539 | 6D | 3.42 | G/A | 0.138 | 4.36 | 0.19 |
|  |  | S6D_6070376 | 6D | 6.07 | T/A | 0.089 | 3.46 | 0.14 |
|  |  | S7A_720744946 | 7A | 720.74 | G/A | 0.233 | 3.02 | 0.14 |
|  |  | S7B_608086910 | 7B | 608.09 | C/A | 0.168 | 3.1 | 0.1 |
|  |  | S7D_4215106 | 7D | 4.22 | G/A | 0.238 | 3.19 | 0.14 |
|  | LS | S1A_14224631 | 1A | 14.22 | C/T | 0.126 | 3.19 | 0.13 |
|  |  | S1B_49723852 | 1B | 49.72 | C/T | 0.178 | 3.22 | 0.13 |
|  |  | S1B_164107163 | 1B | 164.11 | G/C | 0.365 | 3.59 | 0.14 |
|  |  | S1B_592939183 | 1B | 592.94 | C/T | 0.133 | 3.58 | 0.16 |
|  |  | S1D_250212446 | 1D | 250.21 | T/C | 0.292 | 3 | 0.12 |
|  |  | S1D_450556479 | 1D | 450.56 | A/G | 0.107 | 4.45 | 0.15 |
|  |  | S2A_488411487 | 2A | 488.41 | T/C | 0.182 | 3.11 | 0.13 |
|  |  | S2A_748559027 | 2A | 748.56 | T/C | 0.435 | 3.03 | 0.12 |
|  |  | S2D_21561794 | 2D | 21.56 | G/C | 0.293 | 3.61 | 0.15 |
|  |  | S2D_22673899 | 2D | 22.67 | G/C | 0.301 | 3.66 | 0.16 |
|  |  | S3A_21102523 | 3A | 21.1 | T/C | 0.419 | 3.1 | 0.1 |
|  |  | S5A_129333455 | 5A | 129.33 | C/T | 0.285 | 3.09 | 0.15 |
|  |  | S6D_471249189 | 6D | 471.25 | A/T | 0.335 | 4.23 | 0.17 |
|  |  | S7A_268653916 | 7A | 268.65 | T/G | 0.244 | 3.39 | 0.13 |
|  |  | S7A_511720607 | 7A | 511.72 | G/A | 0.184 | 3.52 | 0.11 |
|  |  | S7A_512974235 | 7A | 512.97 | C/T | 0.179 | 3.72 | 0.15 |
|  |  | S7B_700803008 | 7B | 700.8 | A/G | 0.313 | 3.01 | 0.1 |

**Table 5.** Gene hits of marker-trait association (MTAs) regions identified with basic local alignment search tool (BLAST) and their annotation with high confidence by IWGSC assembly BLASTX (NCBI BLAST)

| Trait | Loci | Gene hit | protein | ^a^E-value | ^b^%ID | Protein annotation |
| --- | --- | --- | --- | --- | --- | --- |
| GNS (LS) | S2A_1050029 | TraesCS2B02G020100 | 681aa | 3.90E-15 | 97 | Phenylalanine ammonia-lyase; L-Aspartase-like |
| PH (LS) | S2A_748204192 | TraesCS2D02G532600 | 776aa | 1.90E-06 | 95 | Tetratricopepetide-like helical domain superfamily |
|  |  | TraesCS2D02G532100 | 772aa | 1.90E-06 | 95 | Pentatricopeptide repeat |
| DH (LS) | S5B_586352552 | TraesCS5B02G410800 | 928aa | 8.80E-15 | 100 | Eukaryotic translation initiation factor  Winged helix DNA-binding domain superfamily  Proteasome component (PCI) domain |
|  |  | TraesCS5A02G406000 | 923aa | 8.80E-15 | 100 |  |
|  |  | TraesCS5D02G416000 | 927aa | 3.10E-14 | 97 |  |
|  |  | TraesCS5B02G219100 | 928aa | 5.80E-14 | 94 |  |
|  |  | TraesCS5A02G220000 | 928aa | 5.80E-14 | 94 |  |
|  |  | TraesCS5D02G228100 | 928aa | 5.80E-14 | 94 |  |
| GNS (NS) | S5D_503657305 | TraesCS5D02G459800 | 252aa | 3.60E-12 | 92 | helix-loop-helix (bHLH) domain |
| GY (NS) | S6A_340738287 | TraesCS1A02G176100 | 599aa | 1.00E-10 | 96 | Coiled-coils |
| DH (LS) | S7A_3066534 | TraesCS7A02G023900 | 333aa | 2.80E-07 | 100 | PTHR 44519 |
|  |  | TraesCS4A02G466400 | 381aa | 3.30E-17 | 97 | F-box-like domain superfamily |
| DH (NS) | S7D_6002850 | TraesCS7D02G013900 | 220aa | 3.10E-04 | 100 | Tansmembrane helices; Coild-coils |

Trait legends are same as in table 1; a. E-value: Expect value; b. %ID: Similarity percentage by which sequences are related

**Figure Legends**

**Fig. 1** Pearson's coefficient ( r ) of correlation among traits studied under normal sown (upper right diagonal) and late sown (lower left diagonal) conditions with traits arranged according to first principal component (FPC). The absolute value of 0.189 and 0.184 was found significant for normal sown and late sown trials respectively. **DH**: days to heading, **GFD**: grain filled duration, **PH**: plant height, **SPP**: spikes per plant, **GNS**: grain number per spike, **TKW**: thousand kernel weight and **GY**: grain yield

**Fig. 2** Biplot of studied traits with 125 genotypes under normal sown conditions **(a)** and under late sown conditions **(b)**. PC1 and PC2 are the principal components along x-axis and y-axis respectively. Numbers are genotypes serial number as in additional file 1: Table S1. Trait legends are same as in Table 1.

**Fig. 3** Heat map of squared cosine values of 125 lines in normal and late sown (NS, LS) conditions through factorial analysis along with F1 and F2 on the basis of seven traits

**Fig.4** A panel of 125 lines classified through principal component analysis **(a)** and Agglomerative Hierarchical Clustering **(b)** using 55954 markers

**Fig. 5 a)** Intra chromosomal LD with Mega base (Mb) pair distance annexed with chromosome number at x-axis and inter chromosomal LD **(b)** with number of pairs in significant (p-value <0.05) LD; **c)** LD-decay plot against distance base pair with LOWESS smoothing curve; **d)** Frequency distribution of LD (R^2) shows number of base pairs in significant (p-value <0.05)

**Fig. 6** A Physical map showing significantly associated markers for all the studied traits. Colored shapes indicating markers significantly associated with traits under late sown conditions.

**Fig. 7** Effect of favorable alleles estimated for traits studied under late sown conditions

**Additional Files**

**Additional file 1: Table S1.** Pedigree of 125 studied hexaploid spring wheat lines with PCs, AHC clustering, phenotypic values of studied traits under normal, late sown conditions at three locations, average of three locations under normal, late sown conditions and number of favored alleles in each genotype under late sown conditions.

**Additional file 2: Figure S2.** Single nucleotide polymorphism (SNP) distributions on 21 chromosomes in 125 wheat lines, in the vertical axis are the 21 chromosomes. The horizontal axis shows chromosome length (Mb); 0 ~ 20 depicts SNP density (the number of SNPs per window).

**Additional files 3-9: Figures S3-S9.** Manhattan plot with QQ plot of days to heading, grain filled duration, plant height, spikes per plant, grain numbers per spike, thousand kernel weight and grain yield under normal (DHN, GFDN, PHN, SPPN, GNSN, TKWN, GYN) and late (DHL, GFDL, PHL, SPPL, GNSL, TKWL, GYL) conditions in 125 wheat lines

**Additional file 10: Figure S10.** Temperature details of the cropping season at three experimental locations
